# A MSLA 3D-printing platform for ZIF-8 and UiO-66 membranes: tuning mechanical and adsorption synergy

**DOI:** 10.1039/d6lf00318d

**Published:** 2026-10-22

**Authors:** Amira Nour, Pietro Magarò, Carmine Maletta, Teresa F. Mastropietro, Jesús Ferrando–Soria, Emilio Pardo, Donatella Armentano

**Affiliations:** a Dipartimento di Chimica e Tecnologie Chimiche, Università della Calabria 87030 Rende Cosenza Italy donatella.armentano@unical.it; b Dipartimento di Ingegneria Meccanica, Energetica e Gestionale, Università della Calabria 87030 Rende Cosenza Italy; c Instituto de Ciencia Molecular (ICMOL), Universitat de València Paterna 46980 València Spain

## Abstract

Metal–organic framework (MOF)-based mixed-matrix membranes (MMMs) fabricated by additive manufacturing represent a promising platform for reusable and structurally robust adsorbents for water remediation. Nonetheless, the rational design of such systems necessitates a profound understanding of how MOF incorporation simultaneously affects both structural integrity and functional efficacy, two critical properties for practical application. Herein, masked stereolithography (MSLA) was employed to fabricate 3D-printed MMMs in a single step, incorporating ZIF-8 and UiO-66 MOFs into a commercial photocurable acrylic resin. The influence of MOF incorporation on the structural, mechanical, and adsorption properties of the resulting membranes was systematically investigated, revealing a strong correlation between the intrinsic characteristics of ZIF-8 and UiO-66 fillers and the resulting mechanical and adsorption response of the printed membranes. Nanoindentation measurements showed that UiO-66 acts as a reinforcing filler, increasing the elastic modulus of the printed membranes up to ∼5 GPa, whereas ZIF-8 decreases stiffness and hardness, consistent with a softer and more compliant framework response within the polymer matrix. Despite its lower mechanical reinforcement, the 3D printed ZIF-8-resin provided superior adsorption performance toward brilliant green (BG) dye, a model cationic organic contaminant, maintaining over 95% removal efficiency across five regeneration cycles while preserving its structural stability. In contrast, UiO-66-based membranes exhibited significantly lower removal efficiency (∼37%), highlighting the critical role of MOF surface chemistry and pore accessibility in governing adsorption behaviour. Importantly, the ZIF-8 membranes retained >95% removal efficiency after five adsorption–desorption cycles using ethanol as a mild regeneration solvent, while preserving their structural integrity. These results demonstrate that MSLA printing enables the rational design of regenerable MOF-based membranes with tuneable mechanical and adsorption properties for sustainable water purification applications.

## Abstract 2

Direct MSLA 3D printing of rigid MOF–polymer composites provides precise architectural control and structural durability for functional membrane fabrication. Graphical abstract created with the assistance of Gemini Notebook.

## Graphical abstract

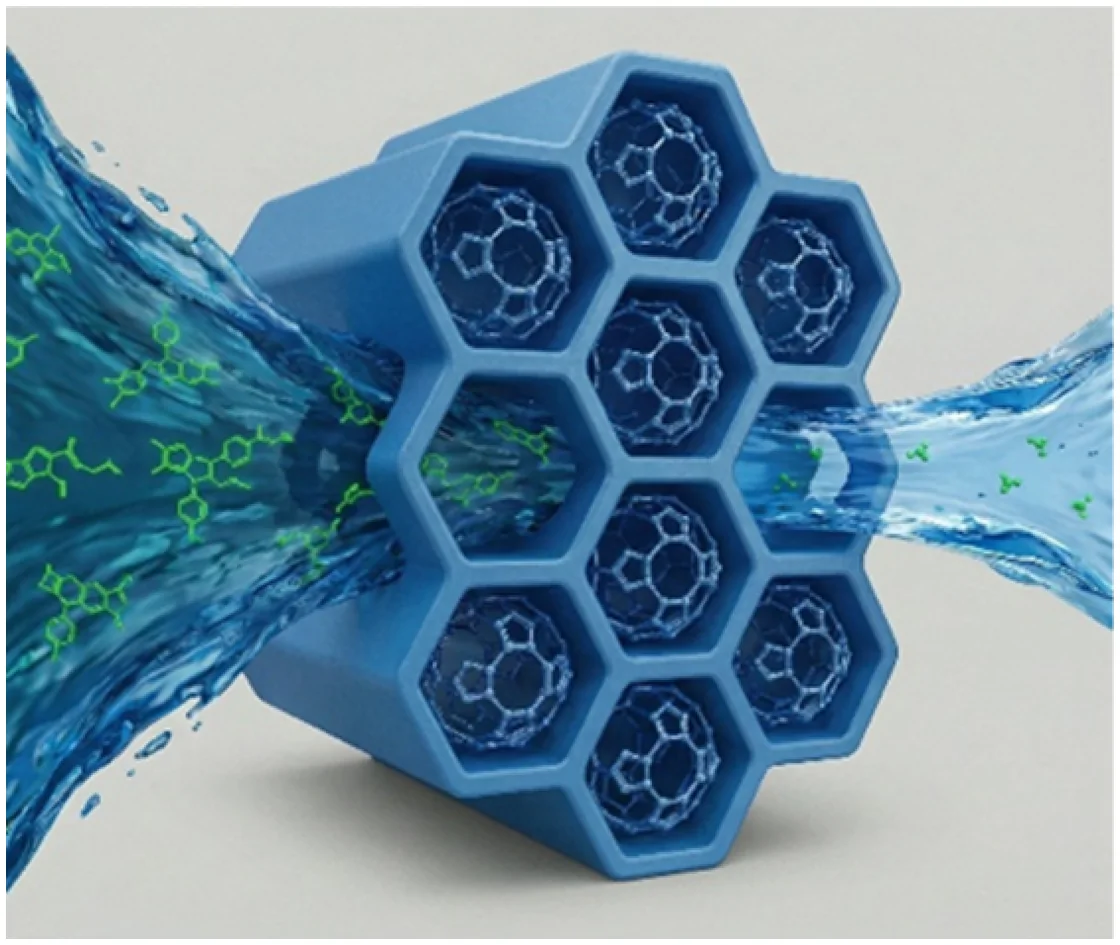

## Introduction

The escalation of global water pollution, driven by the persistent discharge of synthetic organic contaminants into aquatic ecosystems, remains a critical environmental challenge.^1–5^ While adsorption has been widely recognized as a highly effective and operationally straightforward strategy for water remediation due to its inherent versatility, efficiency, and relatively low energy requirements,^6–9^ the practical implementation of typical powder-based adsorbents is hindered by challenges in handling, operational stability and recyclability.^10^ Consequently, there is an urgent need for the development of macroscopic, reusable adsorptive platforms that combine high removal efficiency with structural resilience, as well as scalable manufacturing.

Metal–organic frameworks (MOFs) have attracted considerable attention as advanced adsorbents because of their high surface area, tuneable pore structures, and adjustable pore chemistry.^11,12^ These features enable the efficient adsorption of a broad range of organic and inorganic contaminants. Nevertheless, MOF-based technologies in water treatment systems often require their integration into mechanically stable macroscopic architectures, such as membranes, monoliths, or other composite materials, in order to improve handling, recyclability, and operational durability.^13–15^ In this context, MOF-based mixed-matrix membranes (MMMs) have emerged as promising platforms that combine the adsorption capability of porous crystalline fillers with the mechanical processability of polymeric matrices.^15^

Among the available fabrication strategies, additive manufacturing technologies offer new opportunities for the fabrication of MOF-based MMMs with controlled geometries and tuneable internal architectures.^16–19^ In particular, stereolithography (SLA) and masked stereolithography (MSLA) enable the production of high-resolution structures^18,19^ with reduced material waste, design flexibility, and straightforward digital manufacturing workflows.^20–25^ Recent studies have demonstrated the potential of 3D-printed MOF-containing materials for pollutant adsorption and catalytic remediation processes.^26–29^ However, many reported fabrication strategies still rely on multistep procedures, post-synthetic MOF deposition, or surface-functionalization approaches that may limit scalability and manufacturing simplicity. Moreover, the direct incorporation of preformed MOF particles into photocurable resins represents a simpler and potentially scalable route toward functional adsorptive structures. Nevertheless, maintaining homogeneous dispersion, printability, and preservation of MOF functionality during photopolymerization remains challenging. MSLA was selected here as an accessible desktop vat-photopolymerization platform suitable for directly processing MOF-containing photocurable formulations. Its LCD-based masking system enables simultaneous patterned exposure of each layer, a capability shared with DLP, which instead uses a digital micromirror-based projection system. The practical appeal of MSLA therefore lies in its accessible implementation and suitability for the investigated formulations and membrane geometries. The present study evaluates this fabrication route and the resulting filler-dependent mechanical and adsorption behaviour; it does not establish an intrinsic performance advantage over DLP or other additive manufacturing technologies.

Another critical gap lies in the lack of a systematic correlation between the intrinsic nature of the MOF filler and the dual response of the resulting membrane: mechanical robustness and adsorption efficacy. Indeed, beyond capture performances, the practical deployment of 3D-printed adsorptive membranes also depends critically on their mechanical reliability and long-term structural stability under operational conditions.

During repeated adsorption–desorption cycles, printed structures may experience cracking, deformation, interlayer delamination, or progressive deterioration of their porous architecture, ultimately affecting performance reproducibility and reusability.^30–33^ In photopolymer-based additive manufacturing, the mechanical response of printed composites is governed by a complex interplay between resin formulation, curing conditions, filler dispersion, and interfacial interactions.^32,34,35^ MOF incorporation may further modify local stiffness, viscoelastic dissipation, and defect formation due to differences in framework rigidity, particle–matrix adhesion, and light scattering during curing.^34–38^ As a result, understanding the relationship between MOF incorporation, nano-mechanical behavior,^39^ and adsorption performance is essential for the rational design of reusable and mechanically robust MMMs. Recent studies on advanced functional composites and 3D-printed architectures highlight that material composition, porosity, and filler–matrix interfacial interactions synergistically govern both mechanical load transfer and functional performance,^40–42^ offering valuable insights for tailoring structural integrity alongside site accessibility.

Herein, we report the fabrication of MSLA 3D-printed MOF-based MMMs through the direct incorporation of pre-synthesized ZIF-8 (ref. 43) and UiO-66 (ref. 44) archetypal MOFs into a commercial photocurable acrylic resin. The resulting membranes were systematically investigated to evaluate the filler-dependent effects on structural integrity, nano-mechanical properties, and adsorption performance toward brilliant green (BG) dye as a model organic contaminant. The main objective of this study is the synthesis, MSLA 3D printing, and structural/mechanical evaluation of rigid MOF–polymer composite membranes. Attention was devoted to correlating the intrinsic characteristics of each MOF with the mechanical and adsorptive response of the printed membranes, as well as with their regeneration capability over multiple adsorption–desorption cycles.

## Results and discussion

ZIF-8 and UiO-66 were selected as representative MOF fillers because of their high chemical stability, well-established adsorption capabilities toward organic pollutants,^12^ and distinct structural and mechanical characteristics. In particular, ZIF-8 is characterized by a highly porous and flexible zeolitic framework with large accessible surface area,^45,46^ whereas UiO-66 exhibits a comparatively more rigid zirconium-based framework with excellent structural robustness.^47^ These differences make both MOFs suitable model systems to investigate the influence of filler nature on the mechanical and adsorption behaviour of 3D-printed mixed-matrix membranes (MMMs).

Both MOFs were synthesized on a multigram scale according to previously reported procedures [see SI]. SEM analysis confirmed the formation of homogeneous microcrystalline powders with characteristic morphologies and submicrometric particle size distributions (Fig. 1). ZIF-8 displayed the typical polyhedral morphology with particle sizes in the 200–250 nm range, while UiO-66 consisted of well-defined octahedral crystals with average dimensions of approximately 150 nm.

**Fig. 1:**
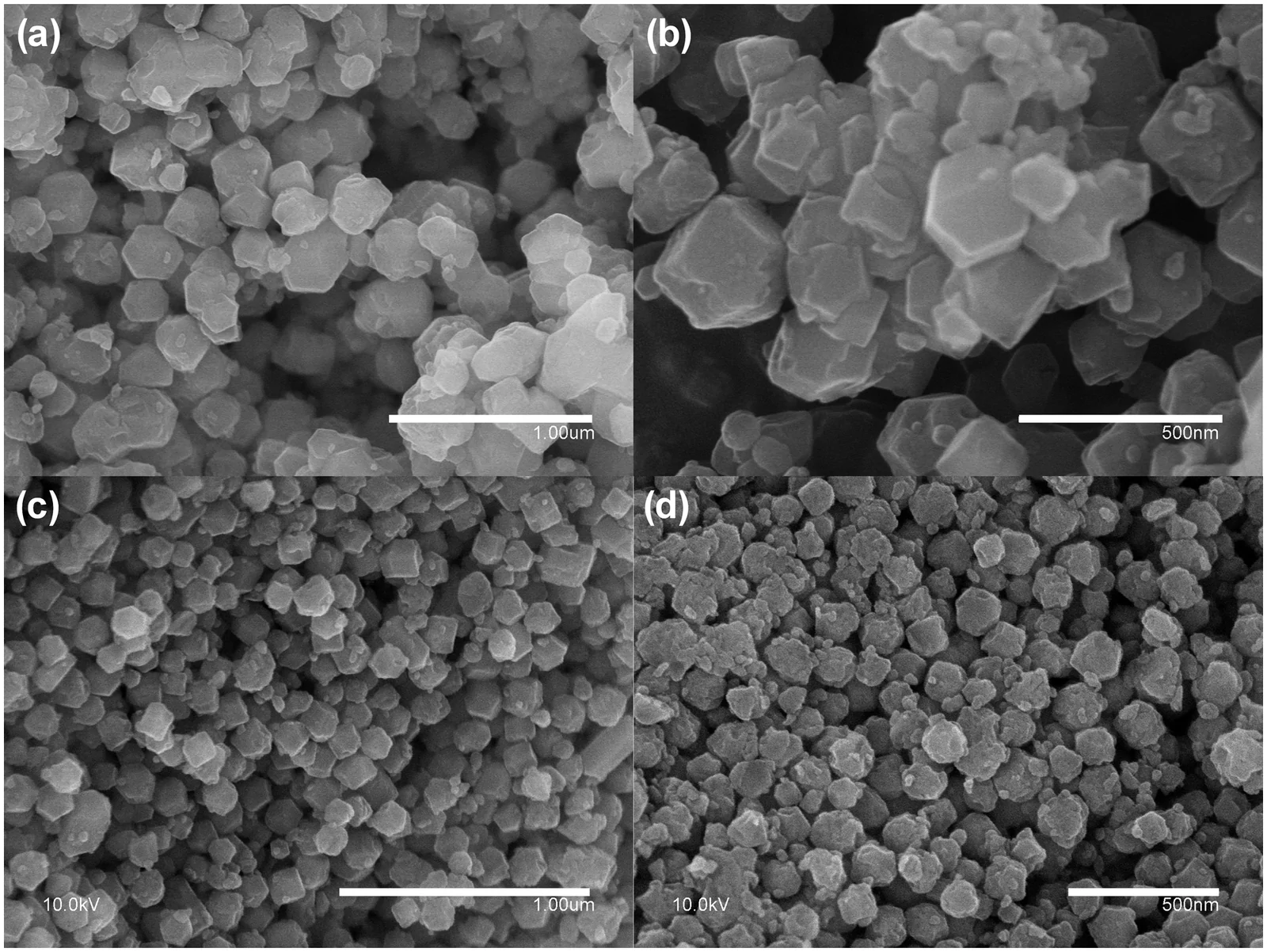
SEM images of ZIF-8 (a and b) and UiO-66 microcrystalline powder samples (c and d) at 1 μm and at 500 nm scale.

### Synthesis and structural characterization of MOFs

Powder X-ray diffraction (PXRD) patterns of the synthesized materials (Fig. 2) matched previously reported data for crystalline ZIF-8 and UiO-66, confirming phase purity and successful framework formation.^48,49^ FTIR spectra further supported the formation of both MOFs through the presence of the characteristic framework vibrations associated with the imidazolate linker in ZIF-8 and the carboxylate-based coordination environment in UiO-66 (Fig. 3 and S1). The spectrum of ZIF-8 is characterized by the distinct vibrations of the imidazolate linker, most notably the sharp C–N stretching peak at approximately 1146 cm^−1^ and various ring stretching and bending modes within the 600–1500 cm^−1^ fingerprint region. Moreover, the sharp peak near 421 cm^−1^ represents the Zn–N stretching vibration mode, confirming the coordination.^42^ For UiO-66, the formation of the framework is supported by the presence of strong absorption bands between 1400 and 1600 cm^−1^, corresponding to the symmetric and asymmetric stretching of the carboxylate groups, as well as Zr–O vibrations observed at lower wavenumbers (around 665 cm^−1^ and 745 cm^−1^).^50^

**Fig. 2:**
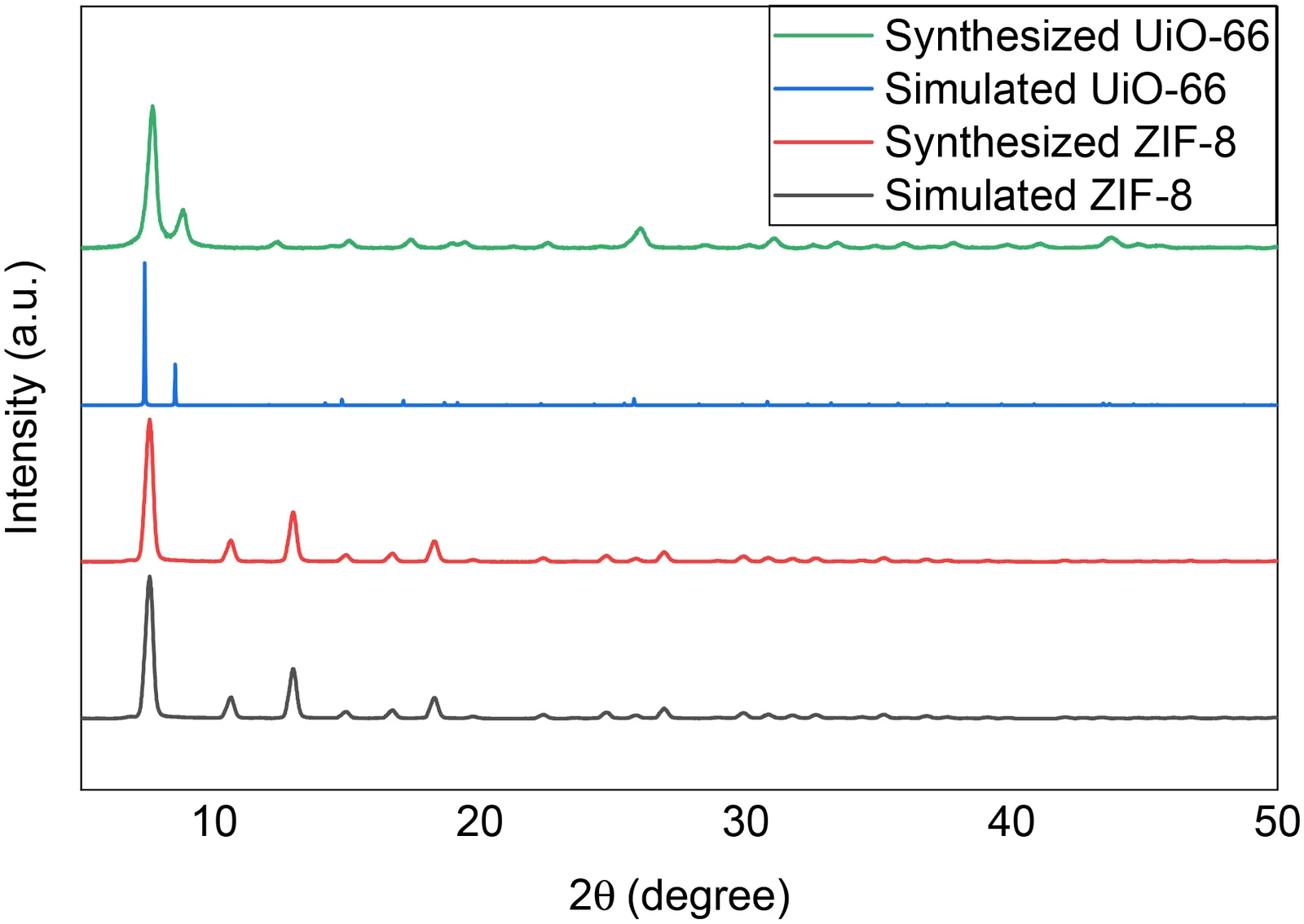
XRD patterns of ZIF-8 and UiO-66 powder samples.

**Fig. 3:**
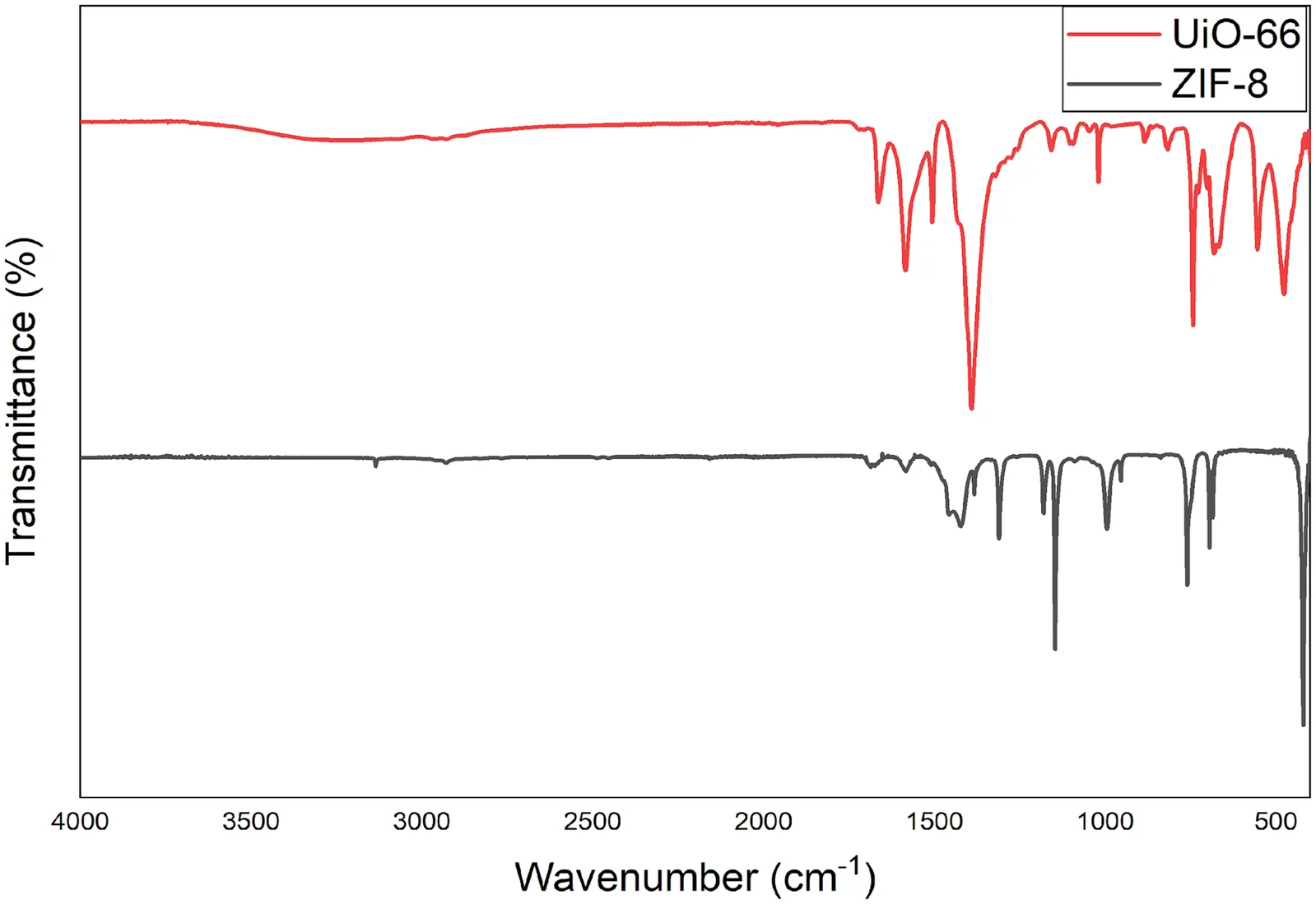
FTIR spectra of ZIF-8 and UiO-66 fillers.

Nitrogen adsorption–desorption measurements revealed the expected microporous behaviour for both materials (Fig. 4). ZIF-8 exhibited the highest BET surface area (1463 m^2^ g^−1^) and pore volume (0.639 cm^3^ g^−1^), while UiO-66 showed lower but still significant porosity values (864 m^2^ g^−1^ and 0.358 cm^3^ g^−1^, respectively) (Table S1). These differences in pore structure and accessible surface area are expected to strongly influence both the adsorption response and the interaction of the MOF particles with the polymer matrix after incorporation into the printed membranes (Table S1).

**Fig. 4:**
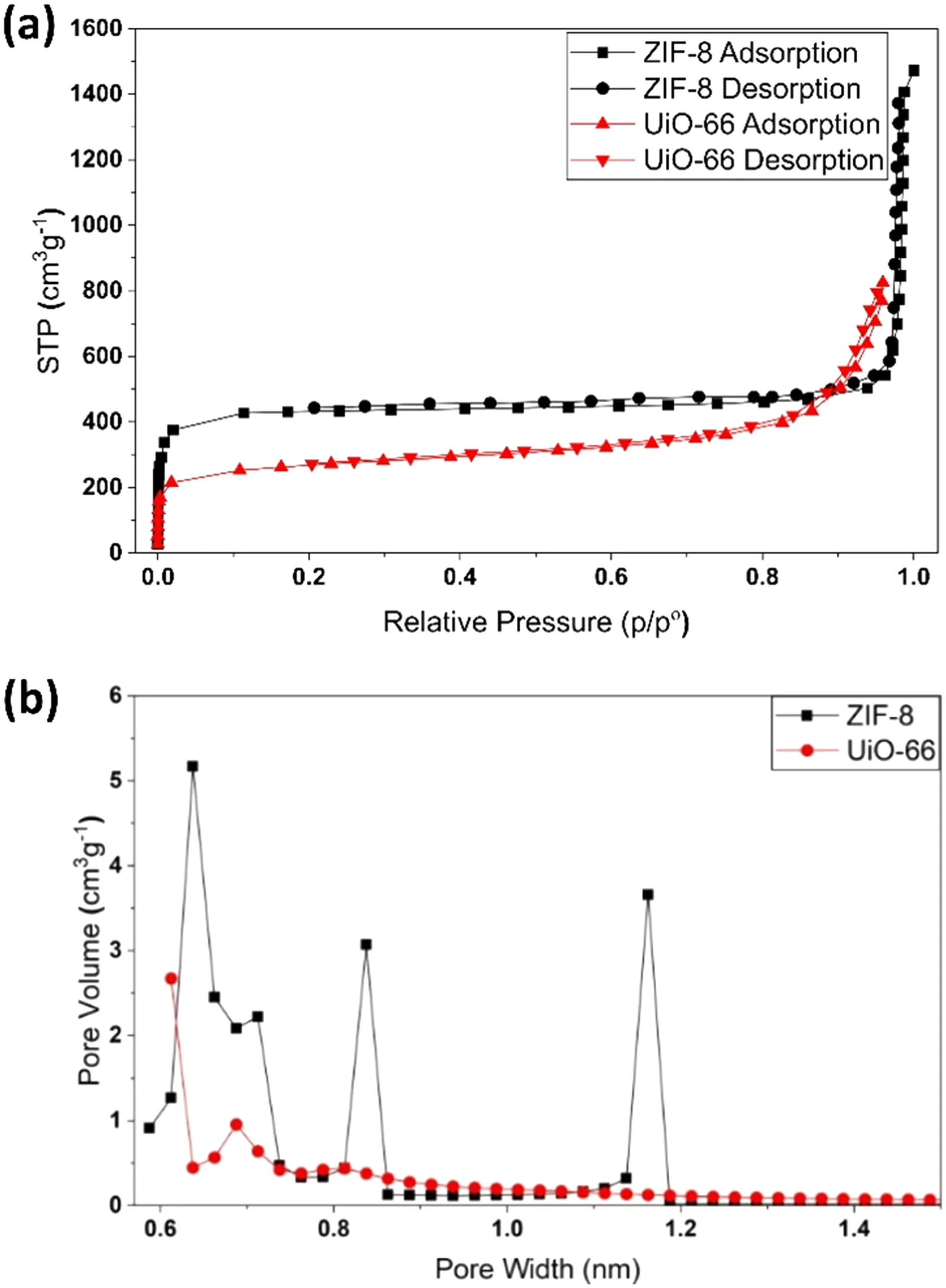
(top) N_2_ adsorption isotherm (77 K) and (bottom) pore size distribution of the activated MOFs.

### MSLA fabrication and structural integration of MOF-based MMMs

The incorporation of porous crystalline fillers into photocurable resins while preserving printability, structural integrity, and MOF functionality represents a key challenge in the fabrication of 3D-printed mixed-matrix membranes. In this work, pre-synthesized ZIF-8 and UiO-66 particles were directly dispersed into a commercial acrylate-based photocurable resin to produce printable suspensions containing 10 wt% MOF loading.

After prolonged stirring under dark conditions to ensure homogeneous dispersion and to prevent premature photopolymerization, the formulations were successfully processed by masked stereolithography (MSLA) without requiring additional surface functionalization, post-synthetic MOF growth, or multistep deposition procedures.

Scheme 1 illustrates the fabrication workflow employed for membrane preparation and post-processing. The membranes were fabricated using an LCD-based MSLA printer with a layer height of 0.05 mm, producing free-standing periodic porous structures with controlled geometry and good dimensional stability (Fig. 5). Because MOF incorporation modifies light transmission within the resin through absorption and scattering effects, exposure conditions were adjusted depending on the filler type to ensure sufficient interlayer adhesion while minimizing excessive UV exposure. After printing, the membranes were washed with isopropanol to remove the uncured resin and post-cured under 405 nm irradiation, yielding mechanically stable MOF-based MMMs.

**Scheme 1:**
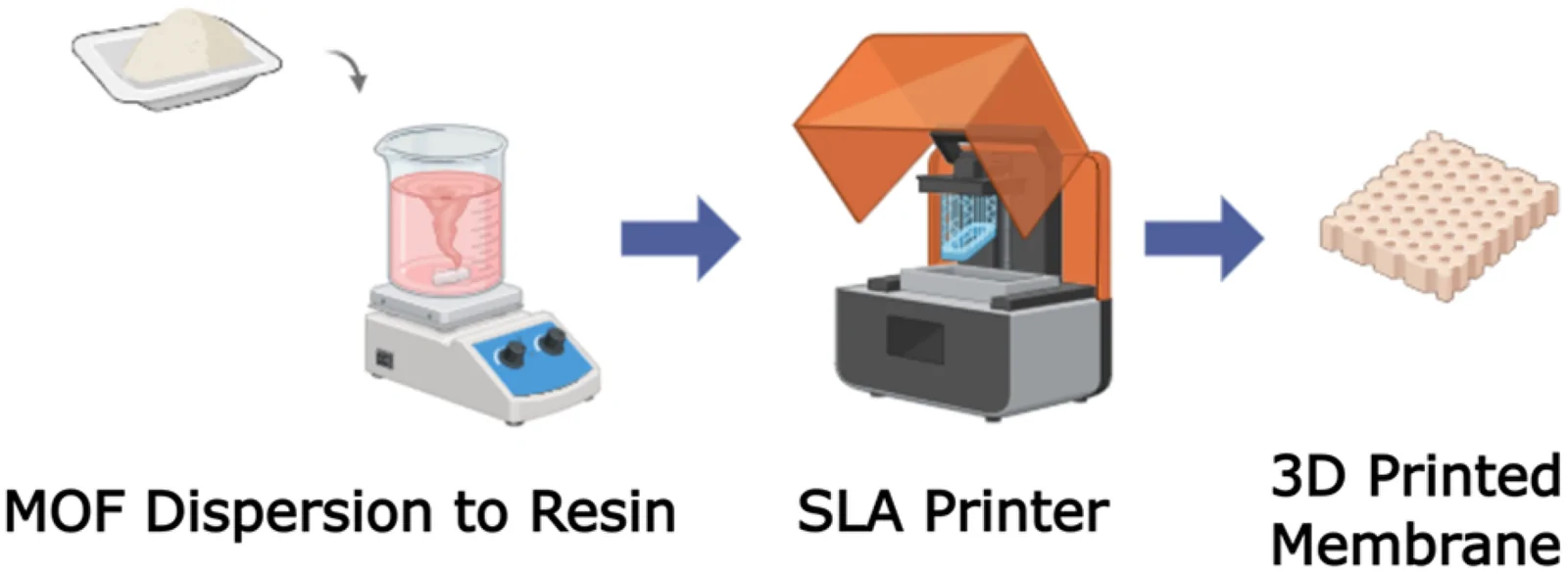
Workflow for the preparation of MOF–resin MMMs and MSLA printing/post-processing steps.

**Fig. 5:**
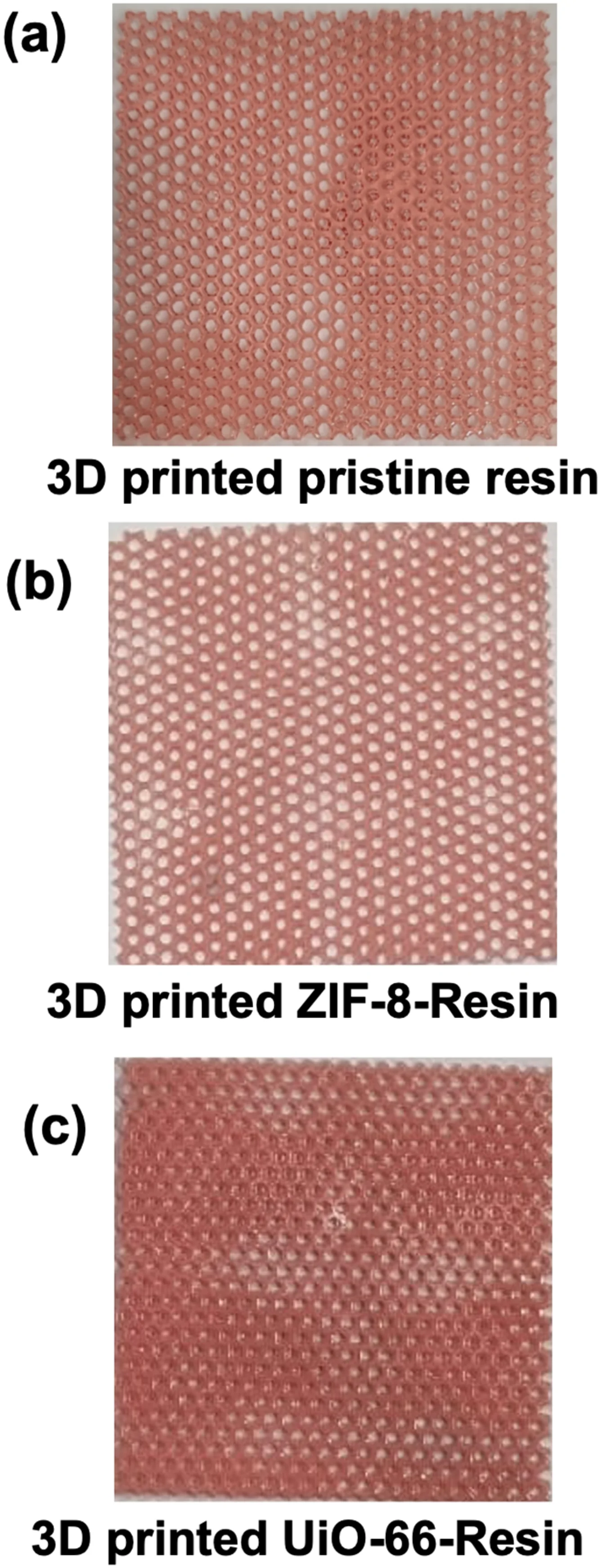
Photographs of representative as-printed membranes [pristine resin (a), ZIF-8 (b) and UiO-66 (c) MOF-loaded formulations].

The morphology and structural integration of the MOFs within the printed membranes were investigated by SEM and EDS analysis. The pristine printed resin displayed smooth surfaces and a homogeneous layered cross-section characteristic of well-cured photopolymer networks (Fig. 6a and b). After MOF incorporation, both composites preserved the overall integrity of the printed architecture. ZIF-8-based membranes exhibited localized surface aggregates, whereas UiO-66 particles appeared more uniformly distributed over the membrane surface (Fig. 6c and e). Nevertheless, cross-sectional SEM images confirmed that both fillers were successfully distributed throughout the membrane thickness (Fig. 6d and f), indicating effective incorporation of the MOFs into the polymer matrix during the printing process. EDS analysis further confirmed the presence of the MOF fillers within the printed composites through the detection of Zn and Zr signals in the ZIF-8- and UiO-66-containing membranes, respectively (Fig. S2). No additional unexpected elemental contributions were observed, supporting the chemical integrity of the printed materials after processing.

**Fig. 6:**
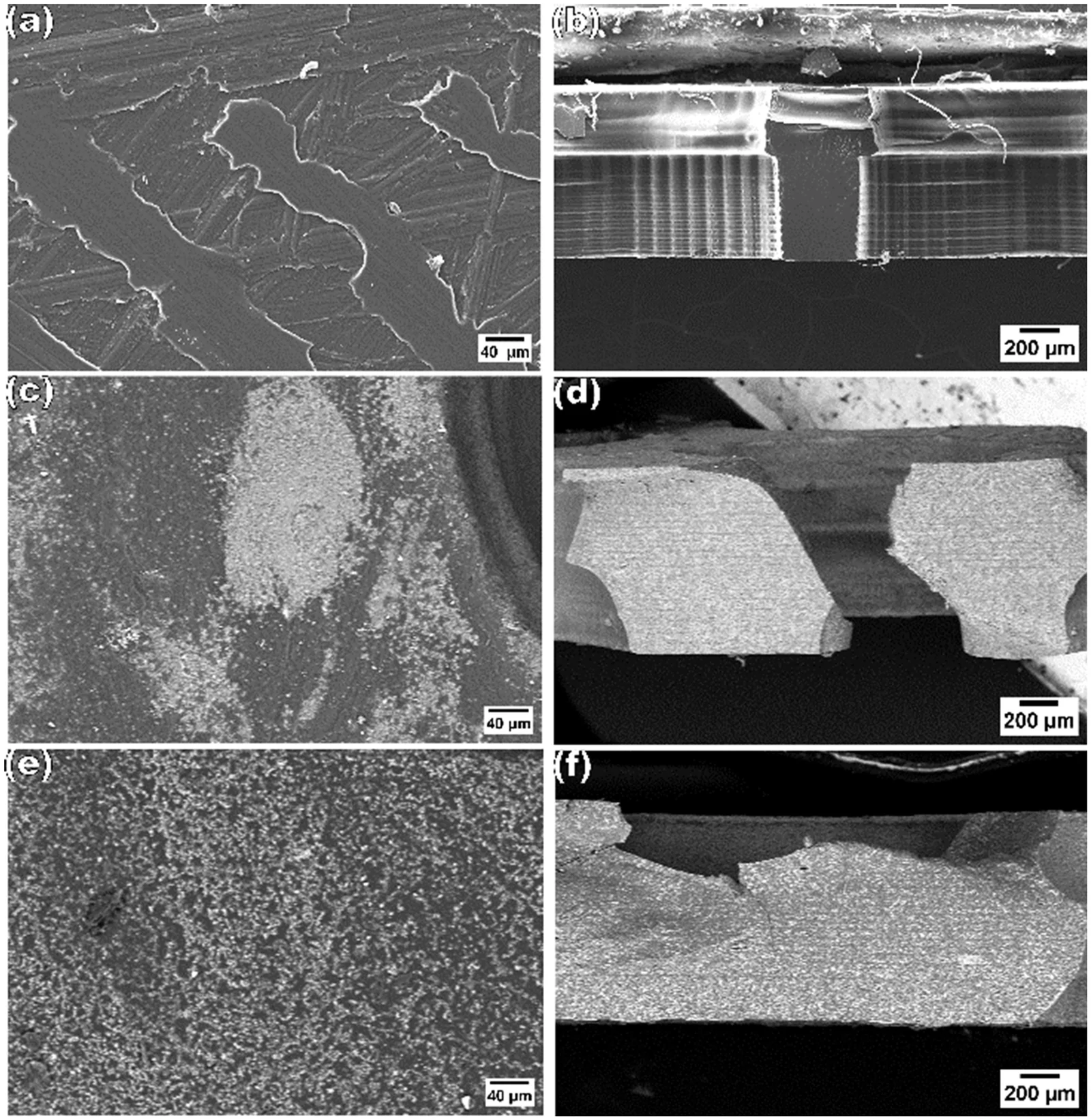
SEM images of 3D printed MOF membranes. Top-view (a, c and e) and cross-section (b, d and f) at 40 and 200 μm scale, respectively: (a and b) pristine resin membrane; (c and d) ZIF-8-resin; (e and f) UiO-66-resin.

The preservation of MOF crystallinity after MSLA printing and post-curing was assessed by PXRD (Fig. 7). The pristine resin membrane exhibited the typical broad amorphous halos associated with the cross-linked acrylate network. In contrast, the characteristic diffraction peaks of both ZIF-8 and UiO-66 remained detectable after incorporation into the printed membranes, confirming that the crystalline frameworks were preserved throughout the printing and curing processes.

**Fig. 7:**
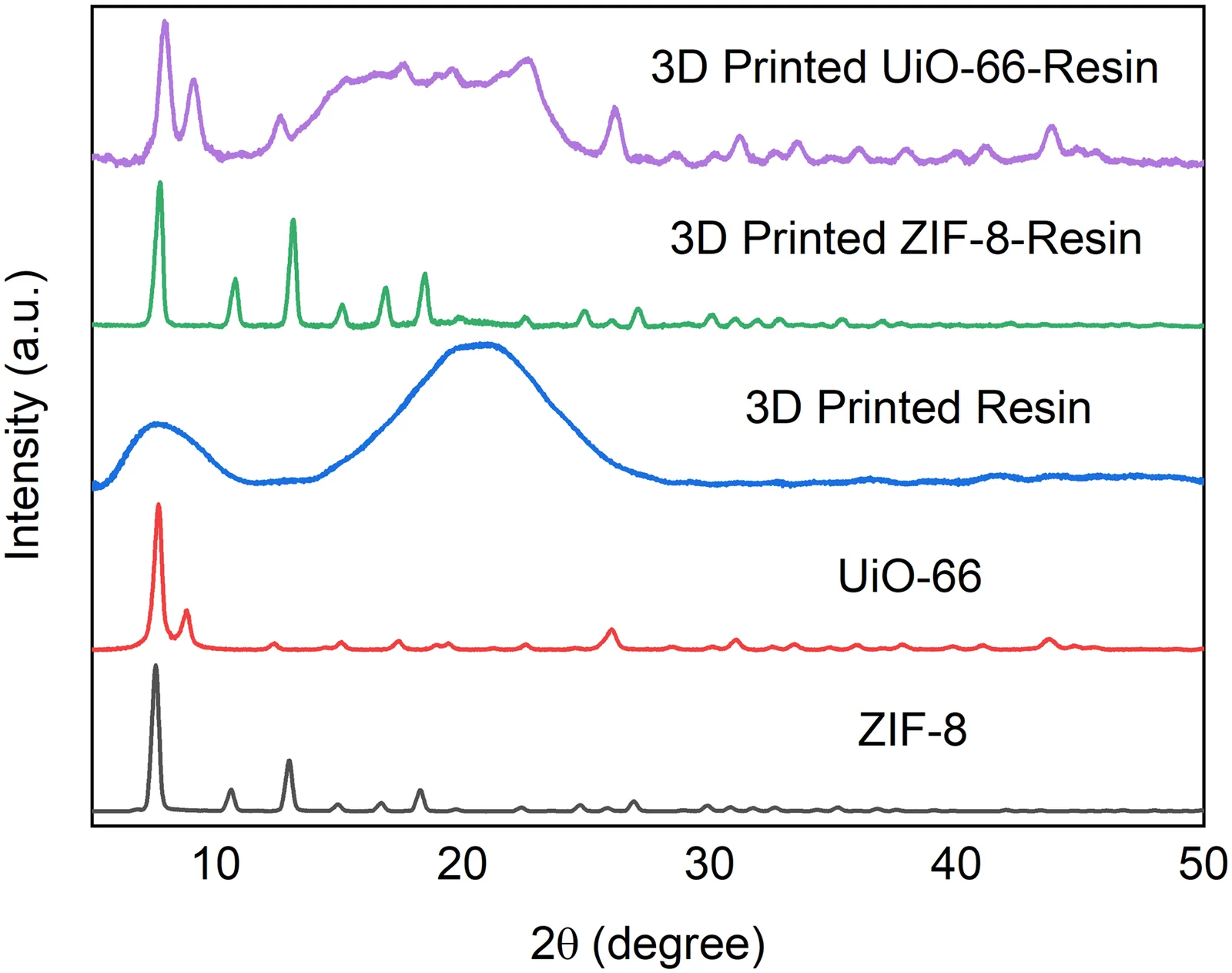
XRD patterns of pristine MOFs and 3D printed membranes.

Notably, the diffraction features of ZIF-8 remained particularly evident within the composite membrane, whereas the UiO-66-based MMM displayed partial superposition of the MOF reflections with the amorphous contribution of the polymer matrix. The apparent absence or complete suppression of the broad amorphous halo of the polymer resin in the 3D-printed ZIF-8-resin composite diffractogram is a well-documented phenomenon in crystalline-filler/polymer hybrid materials.^45^ Highly crystalline frameworks like ZIF-8 exhibit exceptionally sharp and intense Bragg reflections, such as the predominant (110) peak at 2*θ* ≈ 7.3°. When the composite diffractogram is normalized to the maximum intensity of these crystalline peaks, the low-intensity, broad amorphous scattering from the polyacrylate matrix is visually flattened and compressed onto the baseline scale.

FTIR spectra of the printed membranes further confirmed the coexistence of the characteristic vibrational features of both the polymeric matrix and the incorporated MOFs (Fig. 8 and S3). The persistence of the framework-related bands after printing indicates that no significant chemical degradation of the fillers occurred during photopolymerization or post-curing. The spectra for the 3D-printed materials show a superposition of the MOF peaks and the resin's signature, specifically the prominent carbonyl (C=O) stretching peak at 1700 cm^−1^. Notably, no significant shifts in the position of the MOF-specific peaks were observed between the pure powders and the resin composites.^45,50^

**Fig. 8:**
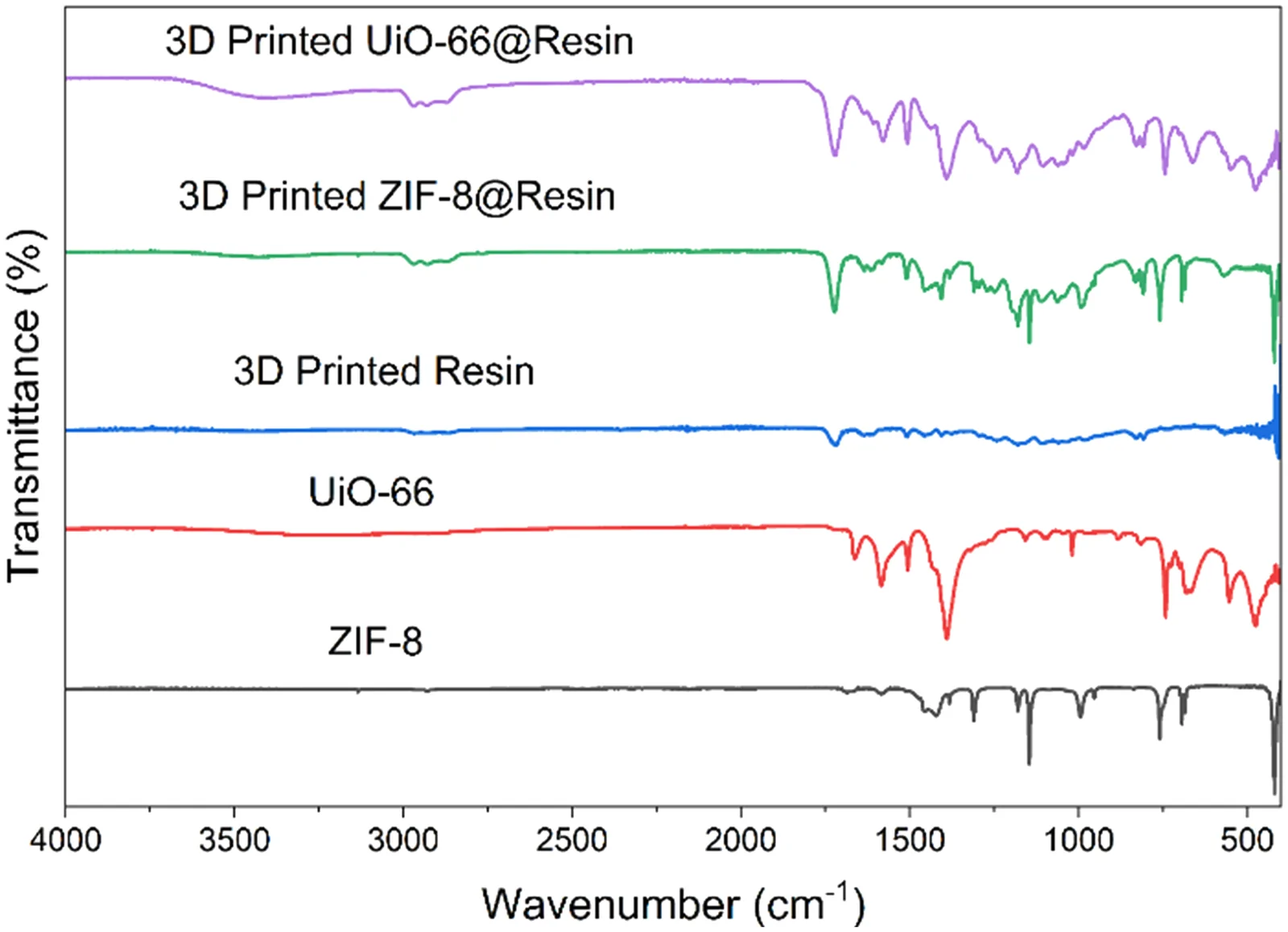
FTIR spectra of MOF powders and 3D printed membranes. Patterns include: ZIF-8 powder (black), UiO-66 (red), 3D printed resin (blue), 3D printed ZIF-8-resin (green), and 3D printed UiO-66-resin (purple).

Overall, these results demonstrate that direct MSLA processing enables the effective integration of preformed MOF particles into photocurable polymer matrices while preserving both structural integrity and functional characteristics of the porous fillers.

### Filler-dependent mechanical behavior of the printed MMMs

Beyond preserving the structural integrity of the MOFs during printing, understanding how filler incorporation affects the mechanical response of the resulting mixed-matrix membranes is essential for their practical implementation in reusable water treatment systems.

In particular, the incorporation of porous crystalline particles into photocurable polymer networks may either reinforce or weaken the matrix depending on the intrinsic rigidity of the filler, particle–matrix interfacial interactions, and the influence of the filler on curing homogeneity and local stress distribution.

The importance of interfacial interactions in multifunctional composite films has also been illustrated by Chen *et al.*, who used hydroxyapatite nanowires to enhance bonding between cellulose fibres and PEDOT, obtaining films combining mechanical robustness with functional properties suitable for applications including solar-driven water evaporation.^51^

The nano-mechanical behaviour of the printed membranes was investigated by sinus mode nanoindentation. Representative load–displacement curves are shown in Fig. 9a. The pristine resin exhibited the characteristic response of a rigid glassy polymer, with relatively limited penetration depths under increasing load. The incorporation of UiO-66 led to a further reduction in penetration depth under comparable loading conditions, indicating enhanced resistance to localized deformation. In contrast, the ZIF-8-based membranes displayed substantially deeper penetration profiles, consistent with a softer and more compliant mechanical response.

**Fig. 9:**
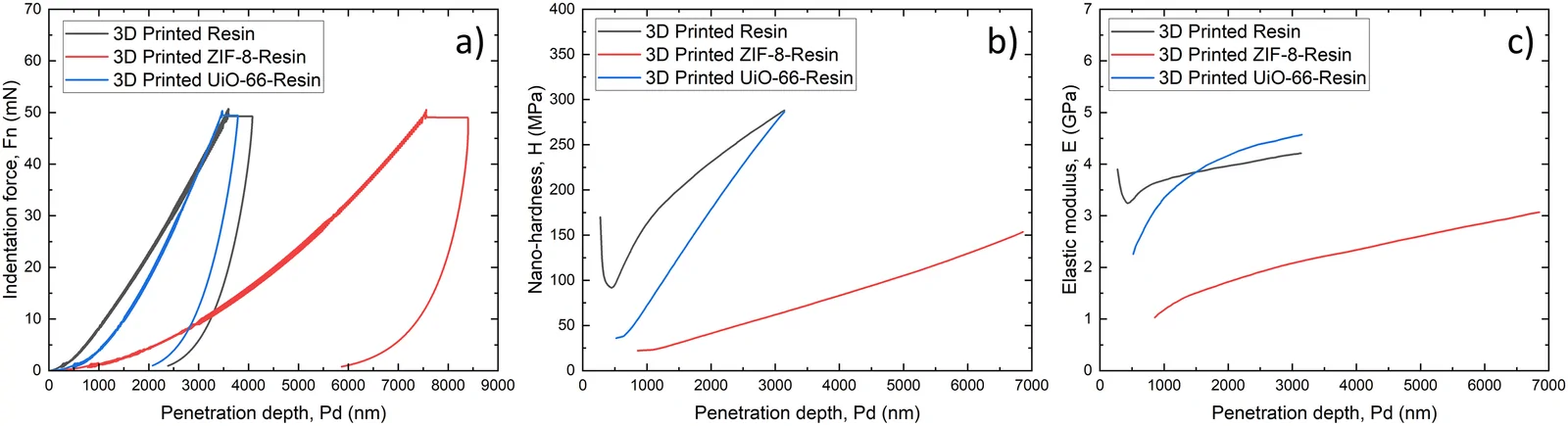
(a) Load–displacement (*F*_n_–*P*_d_) curves obtained from sinus mode nanoindentation of the neat resin and composites containing UiO-66 and ZIF-8. (b) Hardness (*H*) as a function of penetration depth for the neat resin and MOF-reinforced composites. (c) Elastic modulus (*E*) as a function of penetration depth.

The hardness and elastic modulus profiles (Fig. 9b and c) further confirm the distinct mechanical response induced by the two MOF fillers. The pristine resin exhibited elastic modulus values in the 3.5–4.0 GPa range, while UiO-66 incorporation increased the modulus up to approximately 5 GPa. Conversely, the ZIF-8-containing membranes displayed significantly lower elastic modulus values (1–3 GPa), together with reduced hardness across the investigated penetration depth range. Overall, the results indicate that UiO-66 acts as an effective reinforcing filler within the polymer matrix, whereas ZIF-8 induces a marked softening effect.

The distinct mechanical behaviour of the two composites can be rationalized considering the intrinsic characteristics of the corresponding MOF frameworks and their interaction with the surrounding polymer network. UiO-66 possesses a highly connected zirconium-based framework with comparatively high structural rigidity, which likely promotes more efficient stress transfer between filler particles and polymer matrix during indentation. In contrast, the more compliant and highly porous ZIF-8 framework may introduce mechanically weaker regions within the cross-linked resin, acting as localized deformation sites under applied load. Moreover, the localized ZIF-8 aggregation observed by SEM may generate heterogeneous stress distributions and local stress concentrations. Where particle–matrix contact is limited, aggregation may also reduce stress-transfer efficiency, potentially contributing to the lower stiffness observed by nanoindentation and tensile testing. Its contribution cannot, however, be isolated from differences in framework properties, interfacial interactions and possible variations in curing homogeneity.

Dynamic nano-mechanical measurements further support these observations (Fig. 10). The UiO-66-based membranes exhibited the highest storage modulus (*E*′) values, approaching 5 GPa, together with increased loss modulus (*E*″), indicating enhanced stiffness combined with higher energy dissipation capability. In comparison, the ZIF-8 composite displayed substantially lower storage modulus values and a more compliant viscoelastic response throughout the investigated penetration depth range.

**Fig. 10:**
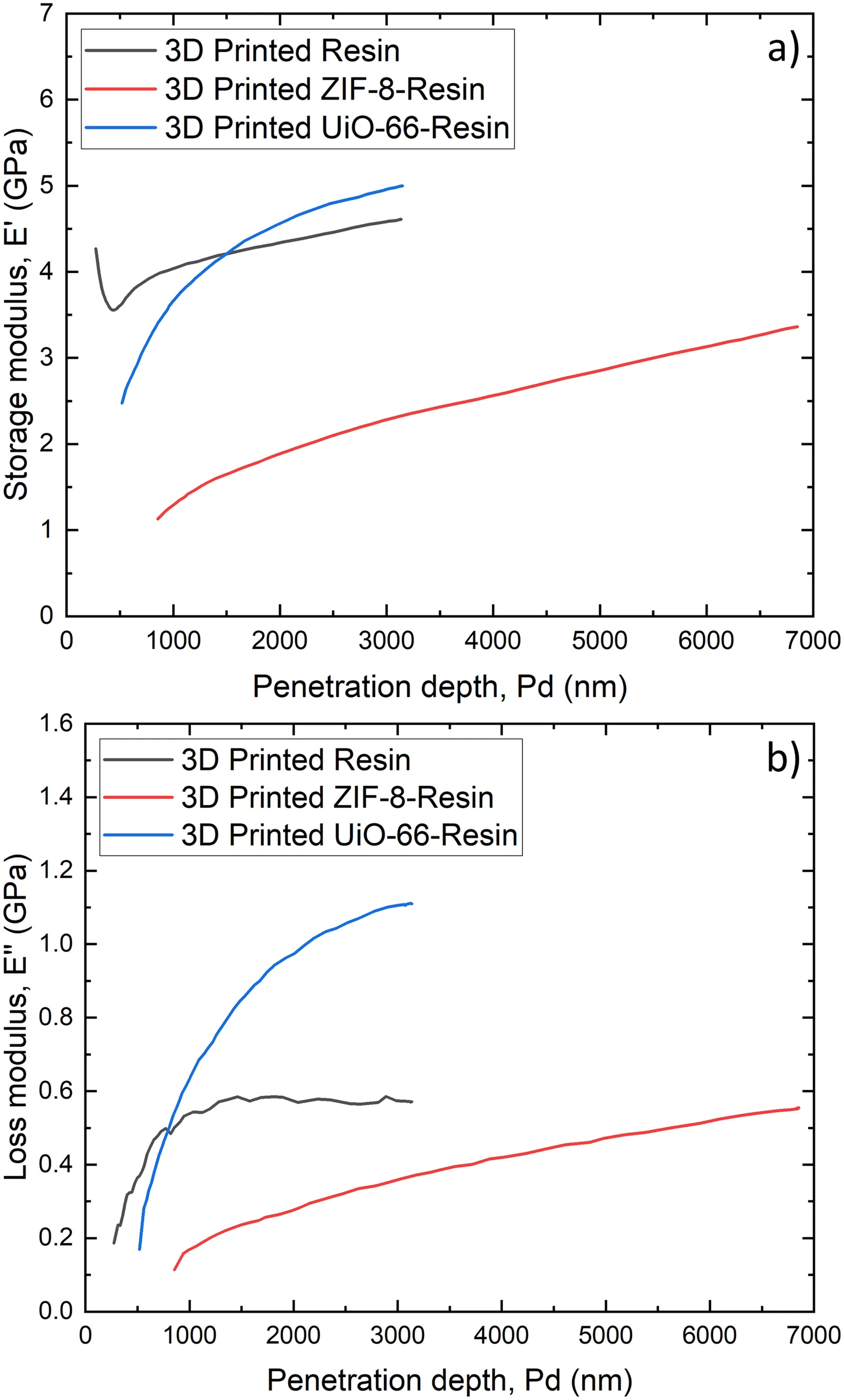
(a) Storage modulus (*E*′) and (b) loss modulus (*E*″) as a function of penetration depth.

To complement the local mechanical characterization obtained by nanoindentation, tensile stress–strain curves were also acquired for the three formulations, as shown in Fig. S6. The corresponding tensile Young's moduli are summarized in Table S5. The neat resin exhibited a modulus of 3.11 ± 0.06 GPa, while the incorporation of UiO-66 increased the modulus to 3.83 ± 0.01 GPa. In contrast, the resin containing ZIF-8 exhibited a lower modulus of 1.54 ± 0.10 GPa. Thus, UiO-66 increased the tensile modulus by approximately 23% relative to the neat resin, whereas ZIF-8 reduced it by approximately 50%.

The tensile results show the same qualitative trend observed by nanoindentation, namely UiO-66/resin > neat resin > ZIF-8/resin. This confirms the reinforcing effect of UiO-66 and the more compliant response induced by ZIF-8. The absolute modulus values obtained from tensile testing and nanoindentation are not expected to be identical because the two techniques probe different deformation conditions: bulk uniaxial deformation in the tensile test and local contact stiffness during indentation. The tensile results for the UiO-66-containing resin should also be interpreted considering that some specimens exhibited printing defects; however, the two analysable specimens provided highly consistent modulus values.

Overall, the nanoindentation and tensile test results demonstrate that the mechanical response of the printed membranes is strongly filler-dependent and closely linked to the intrinsic framework characteristics of the incorporated MOFs. While UiO-66 improves stiffness and viscoelastic resistance, ZIF-8 promotes a softer and mechanically less resistant structure. These differences are particularly relevant for the rational design of reusable adsorptive membranes, where mechanical robustness and adsorption performance must be simultaneously optimized.

### Filler-dependent adsorption behaviour of the printed MMMs

The adsorption performance of the printed membranes was evaluated using brilliant green (BG) as a model cationic organic contaminant. BG is a hydrophobic triarylmethane dye widely employed in industrial and biomedical applications and is considered an environmentally relevant pollutant because of its persistence and toxicity in aquatic environments.^52^ The adsorption experiments were performed by immersing the printed membranes in unbuffered aqueous BG solutions (5 ppm, 50 mL) and monitoring the dye removal efficiency as a function of time (Fig. 11 and S4 and Table S2). Evaluating BG capture at 5 ppm and ambient pH ∼5.0 served as a functional proof-of-concept to confirm that the embedded MOF fillers remain active and accessible within the photopolymerized matrix (see SI). The pristine printed resin exhibited only limited adsorption capability, reaching approximately 9% BG removal at equilibrium, which is consistent with the absence of accessible porous adsorption sites within the cross-linked polymer matrix. In contrast, the incorporation of MOFs significantly enhanced the adsorption response of the printed membranes, although with markedly different behaviours depending on the filler nature.

**Fig. 11:**
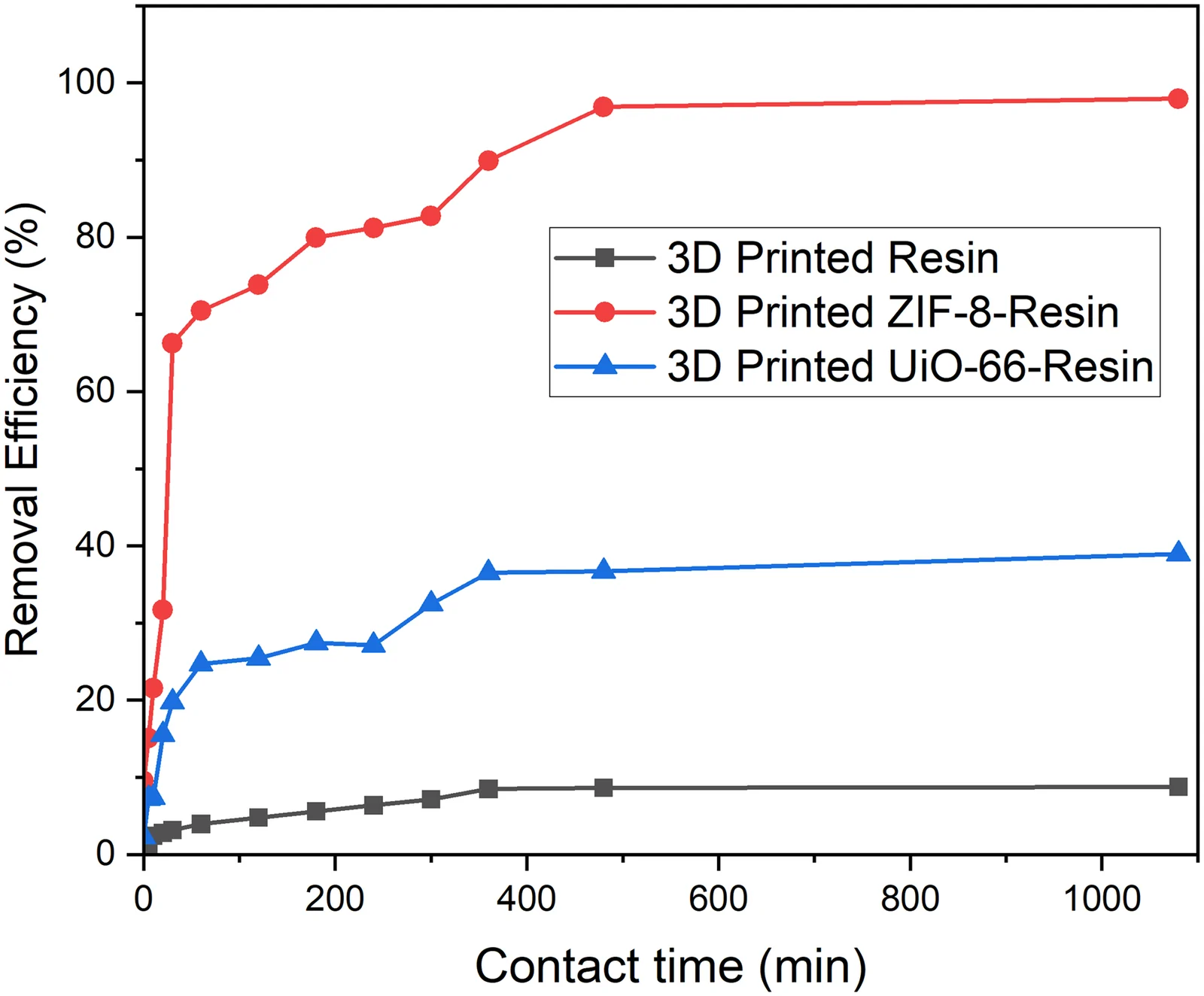
Time-dependent removal efficiency of BG dye using 3D printed membranes (data in Table S2): pristine resin, ZIF-8- resin, and UiO-66-resin. Initial dye concentration was 5 ppm. (Graphics are organized from data reported in Table S2.) Each experiment was performed in triplicate, and results are reported as average values ± 3 SD.

Among the investigated systems, the ZIF-8-based MMMs displayed the highest adsorption efficiency and fastest uptake kinetics. Approximately 80% BG removal was achieved within 200 min, increasing to ∼90% after 360 min and approaching complete removal at equilibrium. In comparison, the UiO-66-based membranes exhibited substantially lower BG removal efficiencies, reaching approximately 37% under identical conditions. The superior adsorption performance of the ZIF-8 composite can be attributed to the high accessible surface area and pore volume of the framework (see Table S1), together with the favourable interaction of BG molecules with the ZIF-8 surface through combined electrostatic, hydrophobic, and π–π interactions.^52^ Because brilliant green (BG) has an approximate kinetic diameter of ∼1.0 nm, its diffusion into the internal micropores of ZIF-8 (average pore width 0.77 nm, Table S1; pore window ∼0.34 nm) is sterically hindered. Consequently, dye capture by ZIF-8 is predominantly governed by interactions at surface-exposed active sites, crystal edge defects, grain boundaries, and hierarchical interparticle voids within the composite.^53^

Importantly, the high adsorption efficiency observed despite the partial aggregation of ZIF-8 particles indicates that a significant fraction of the adsorbing active sites remains accessible after incorporation into the printed polymer matrix.

Conversely, although UiO-66 possesses good chemical stability, larger pore width, and a more versatile range of electrostatic and hydrogen bonding interactions, a lower accessible surface area and less favourable surface interactions toward BG likely limit its adsorption performance.^54^ Hence, dye capture by the 3D-printed MOF membranes operates through a multi-modal cooperative mechanism combining physical and chemical forces, consistent with recent composite membrane studies.^55,56^ Physisorption occurs *via* π–π stacking between the aromatic rings of BG and the imidazolate linkers of ZIF-8 or benzene rings of UiO-66, as well as hydrophobic interactions with the polymer matrix. Concurrently, electrostatic interactions (chemisorption-like behavior) might occur between the cationic BG^+^ species and the polar carbonyl groups of the acrylate matrix and nitrogen donor sites of ZIF-8.

The kinetic analysis revealed different rate-controlling behaviours for the three investigated systems (data in Table S3), which comply with our previous hypothesis. The pseudo-second-order model provided the best fit to the adsorption kinetics of the pristine polyacrylate resin.

For the UiO 66-containing membrane, the adsorption profile was best described by the intraparticle diffusion model. It showed approximately zero intercept, suggesting that intraparticle diffusion is the predominant rate-limiting step governing the adsorption process.^50^ For the ZIF-8-containing membrane, both the pseudo-first-order and the intraparticle diffusion models provided a satisfactory description of the experimental data, with the intraparticle diffusion model showing a slightly better correlation. This finding may suggest that the adsorption process involves multiple mass-transfer steps, with both initial surface adsorption and diffusion through the microporous ZIF 8 network potentially contributing to the overall kinetics. These results highlight the strong dependence of adsorption behaviour on the intrinsic framework characteristics of the incorporated MOF, including pore accessibility, surface chemistry, and adsorbate–framework interactions. Interestingly, the adsorption results reveal the opposite trend compared to the mechanical response of the printed MMMs. While UiO-66 acted as an effective reinforcing filler but exhibited moderate adsorption performance, ZIF-8 promoted significantly higher adsorption capability at the expense of reduced mechanical stiffness. This behaviour emphasizes the existence of a filler-dependent trade-off between mechanical reinforcement and adsorption accessibility in MOF-based printed composites.

Confocal laser scanning microscopy (CLSM) images collected before and after adsorption experiments further support the contribution of the MOFs to dye uptake (Fig. S5). The pristine resin displayed relatively smooth surfaces with limited visible changes after exposure to BG solution, whereas the MOF containing membranes exhibited darker and more heterogeneous regions after adsorption, consistent with localized dye accumulation associated with the embedded porous fillers.

Table 1 compares the adsorption performance of the developed MOF-based MMMs with previously reported 3D-printed adsorptive materials described in the literature. Although direct comparisons remain inherently difficult because adsorption performance strongly depends on experimental conditions, adsorbate concentration, membrane geometry, and operational parameters, the ZIF-8-based printed membranes developed in this work exhibit adsorption efficiencies comparable to those reported for state-of-the-art 3D-printed adsorptive systems. On the other hand, although UiO-66 has demonstrated excellent adsorption performance toward cationic dyes, reports on 3D-printed UiO-66 containing membranes for dye adsorption remain extremely limited, leaving unanswered questions regarding the preservation of adsorption functionality.

**Table 1:** Comparison of the performance of the 3D-MOF-resin membrane with different 3D-printed adsorbents

Adsorbent	Printing method	MOF loading (wt%)	Membrane size (mm)	Contact time (min)	Volume (mL)	*C*_0_ (ppm)	Dye	*R* (%)	Ref.
3D-HPC@MIL-100(Fe)	DIW	—	15 × 15 × 5	10	10	20	MB	98.34	57
							RhB	96.93	
							CV	97.43	
							MG	98.99	
3D-HPC@HKUST-1	DIW	—	15 × 15 × 5	10	10	20	MB	83.79	57
							RhB	98.24	
							CV	98.51	
							MG	99.68	
Cu-MOFs/PLA	FDM	—	—	10	10	10	MG	>90	27
ZIF-8@ZnO-NPs/ABS	FDM	10	14 × 14 × 9.5	300	5	5	MG	91	58
MOF-MMC	SLA	12.5	8 × 8 × 5.5	20	50	5	RhB	97–100	26
							MG		
3D-MOF@clay	DLP	—	18 × 18 × 12	60	50	60	MO	80.5	59
				60	50	60	DR31	58.7	
				60	50	60	MG	8.8	
				60	50	60	RhB	13.97	
HKUST-1/PLA–PEG	FDM	50	15 × 15 × 9	720	2	18	MB	93.4	60
MXene/ZIF-8	DLP	10	18 × 18 × 12	60	50	30	DR31	91.98	61
							MO	84.9	
PDA/MOFs/MXene/3DS	DLP	—	Ø19 × 10 mm	120	20	20	CR	99.13	62
Cu-BTC/CA-GE	DIW	13	Hexagon, 5 mm height	20	50	20	MB	99.8	63
ZIF-8-resin	SLA	10	12 × 12 × 1	360	50	5	BG	89.93	This work
UiO-66-resin	SLA	10	12 × 12 × 1	360	50	5	BG	36.54	This work

Importantly, the present approach combines direct incorporation of pre-synthesized MOF particles, straightforward MSLA fabrication, and effective membrane regenerability within a single integrated platform. To the best of our knowledge, this study also represents the first report describing the use of MSLA-fabricated MOF-based mixed-matrix membranes for brilliant green removal from aqueous solutions. Overall, the results demonstrate that direct MSLA incorporation of MOF particles enables the fabrication of functional adsorptive membranes with tunable adsorption behavior, strongly governed by the physicochemical characteristics of the selected framework.

It is important to emphasize that the commercial photocurable acrylate-based resin, which constitutes 90 wt% of the fabricated hybrid membranes, serves a vital role beyond acting as a simple structural binder. The highly cross-linked polyacrylate network provides the critical dimensional fidelity, structural integrity, and mechanical robustness required to preserve complex 3D-printed architectures during and after stereolithography.

Furthermore, the chemical composition of the polyacrylate matrix actively governs the spatial distribution, aggregation behaviour, and interfacial stability of the embedded MOF particles. The abundance of polar functionalities (such as ester and carbonyl groups) inherent to the acrylate monomers can participate in favourable non-covalent or dipole–dipole interactions with the external surfaces of the ZIF-8 and UiO-66 frameworks. These interfacial interactions are essential for mitigating severe particle macro-phase separation, improving filler-matrix adhesion, and ultimately dictating the accessibility of the underlying microporous channels for successful wastewater remediation applications.

### Regeneration and structural stability of the ZIF-8 MMMs

For practical water remediation applications, adsorptive membranes must retain both adsorption performance and structural integrity over repeated operational cycles. Therefore, the regeneration capability of the ZIF-8-based mixed-matrix membranes was investigated through consecutive adsorption–desorption experiments using different regeneration solvents.

After each adsorption cycle, the membranes were immersed in either acetone or ethanol under static conditions to promote desorption of the adsorbed brilliant green molecules, followed by water washing and thermal drying prior to reuse. The adsorption efficiencies measured over five (up to ten) consecutive regeneration cycles are summarized in Fig. 12 (or Fig. S7) and Table S4.

**Fig. 12:**
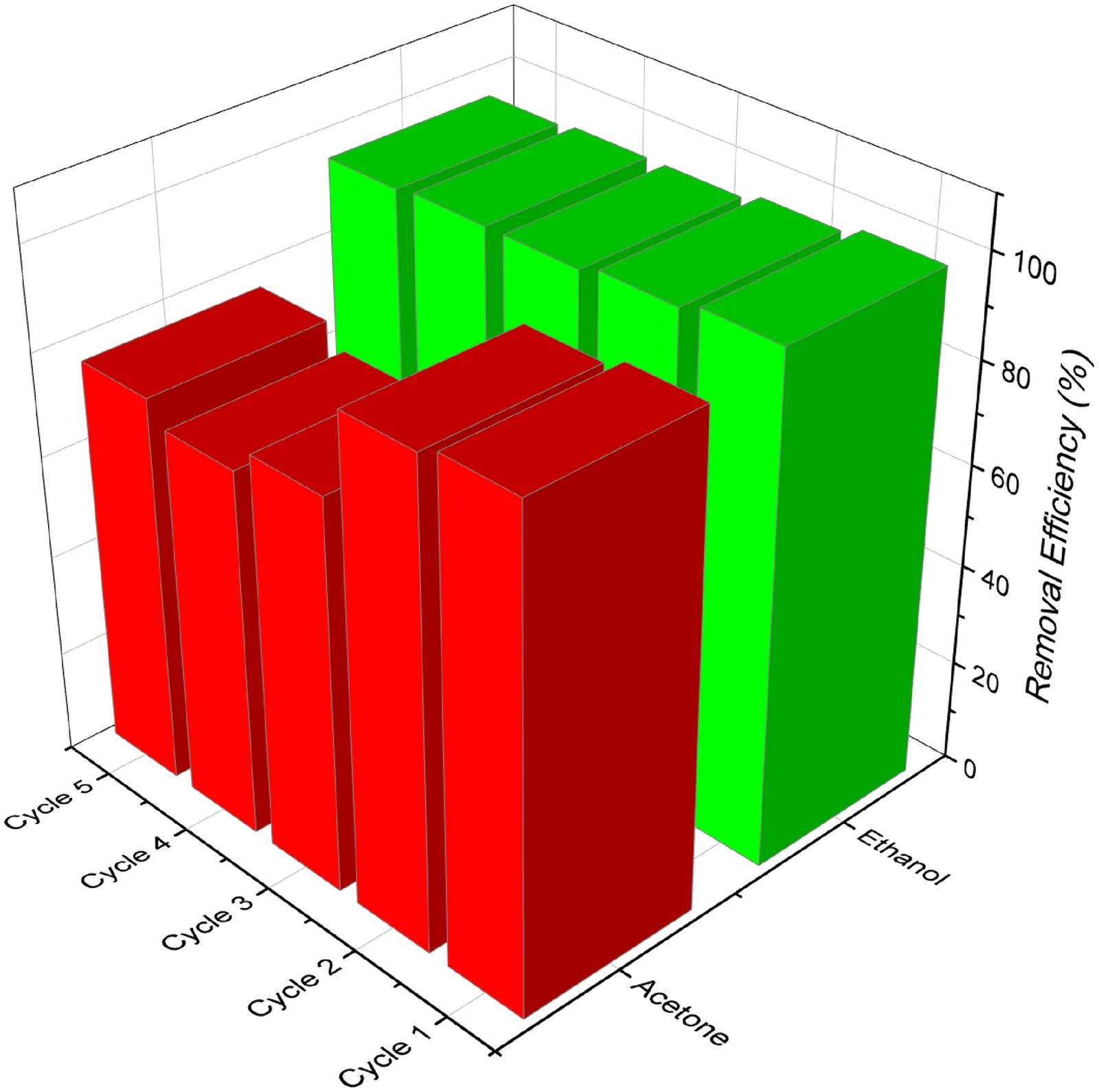
Regeneration performance of the 3D printed ZIF-8-resin membrane for BG removal (5 ppm, 50 mL) over multiple cycles. Comparison between washing with acetone and ethanol as regenerants.

The ZIF-8 MMMs maintained high adsorption performance throughout the repeated cycles, although the regeneration behaviour strongly depended on the selected solvent. When acetone was employed, the BG removal efficiency progressively decreased from 96.2% during the first cycle to 76.3% after the fifth cycle, suggesting partial loss of accessible adsorption sites and/or incomplete dye desorption over repeated operation. In contrast, ethanol-treated membranes retained removal efficiencies above 95% even after five cycles, demonstrating more stable long-term adsorption performance.

The superior behaviour observed with ethanol likely arises from its effective desorption capability combined with lower aggressiveness toward the polymeric matrix compared to acetone. Importantly, no visible cracking, deformation, or macroscopic deterioration of the membranes was observed after the regeneration experiments, indicating good mechanical stability of the printed structures under repeated operational conditions.

The structural integrity of the regenerated membranes was further confirmed by PXRD and SEM analyses (Fig. S8 and S9). SEM images revealed that the overall membrane morphology and distribution of ZIF-8 particles within the polymer matrix were largely preserved after repeated use, without evidence of significant particle leaching or large-scale aggregation. Likewise, the characteristic diffraction peaks of ZIF-8 remained detectable after the regeneration cycles, confirming preservation of the crystalline framework during repeated adsorption–desorption operation. Furthermore, the surrounding polymeric matrix likely plays a protective role, safeguarding the structural integrity of the embedded MOF framework under aqueous conditions. The sustained removal efficiency (>94%) across multiple ethanol regeneration cycles (Fig. 12 and S7) serves as a functional benchmark, indirectly proving that the chemical stability and porous architecture of the MOF filler remain fully preserved inside the 3D composite network during operational water treatment.

Overall, these results demonstrate that the direct MSLA-fabricated ZIF-8 MMMs combine high adsorption efficiency with good structural durability and regeneration capability, highlighting their potential as reusable adsorptive membranes for sustainable water treatment applications. While performance across 5–10 consecutive cycles confirms robust short-to-medium-term reusability, evaluating long-term durability over extended operational cycles remains a key focus of ongoing work.

## Conclusions

In this work, MOF-based mixed-matrix membranes (MMMs) were successfully fabricated by masked stereolithography (MSLA) through the direct incorporation of pre-synthesized ZIF-8 and UiO-66 particles into a commercial photocurable acrylic resin. The adopted approach enabled the production of mechanically stable and structurally well-defined porous membranes without requiring post-synthetic MOF growth or multistep surface functionalization procedures, highlighting the potential of MSLA printing as a powerful route for designing next generation functional adsorptive architectures.

The results revealed a pronounced filler-dependent influence of the incorporated MOFs on both the mechanical and adsorption behaviour of the printed membranes. Nanoindentation measurements showed that the rigid UiO-66 framework acts as an effective reinforcing filler, increasing the stiffness and viscoelastic resistance of the polymer matrix, whereas ZIF-8 induces a softer and more compliant mechanical response. Conversely, ZIF-8-based MMMs exhibited significantly superior adsorption performance toward brilliant green dye, achieving near-complete removal at equilibrium, while UiO-66-based membranes displayed substantially lower adsorption efficiency under identical conditions.

These opposite trends highlight the existence of a critical filler-dependent trade-off between mechanical reinforcement and functional efficiency in MOF-based printed composites, governed by the intrinsic structural and surface characteristics of the selected framework. Importantly, the ZIF-8 membranes retained high adsorption efficiency over multiple adsorption–desorption cycles using ethanol as a mild regeneration solvent, while preserving their structural integrity and crystallinity.

Overall, this study demonstrates that direct MSLA processing combined with a rational selection of MOF fillers provides an effective platform for the rational design of high performance, regenerable and mechanically reliable MOF-based MMMs with tunable structure–property relationships. The combination of additive manufacturing, accessible processing, and filler-dependent functional tuning offers promising opportunities for the development of reusable adsorptive materials for sustainable water remediation applications.

## Conflicts of interest

There are no conflicts to declare.

## Supplementary Material

LF-OLF-D6LF00318D-s001

## Data Availability

The data supporting this study are available in the supplementary information (SI), including Fig. S1–S9, Tables S1–S5, and the experimental data. Additional information is available from the corresponding authors upon reasonable request. Supplementary information is available. See DOI: https://doi.org/10.1039/d6lf00318d.
