# Supplementary material for: A MSLA 3D-printing platform for ZIF-8 and UiO-66 membranes: tuning mechanical and adsorption synergy
Source: RSC Appl Interfaces. 2026 Oct 22. Online ahead of print. doi: 10.1039/d6lf00318d (PMC13749482; doi:10.1039/d6lf00318d)
Supplement: Supplementary file 1 [file LF-OLF-D6LF00318D-s001.pdf]

Supplementary Information (SI) for RSC Applied Interfaces.
This journal is © The Royal Society of Chemistry

Supporting Information

A MSLA 3D-Printing Platform for ZIF-8 and UiO-66 Membranes: Tuning Mechanical and Adsorption Synergy.

Amira Nour,[a] Pietro Magarò,[b] Carmine Maletta,[*, b] Teresa F. Mastropietro,[*,a] Jesús Ferrando–Soria,[c] Emilio Pardo[*, c] and Donatella Armentano[*, a].

[a] Dipartimento di Chimica e Tecnologie Chimiche, Università della Calabria. 87030, Rende, Cosenza, Italy.

[b] Dipartimento di Ingegneria Meccanica, Energetica e Gestionale, Università della Calabria. 87030, Rende, Cosenza, Italy.

[c] Instituto de Ciencia Molecular (ICMOL). Universitat de València Paterna 46980, València (Spain)

E-mail: carmine.maletta@unical.it; teresafina.mastropietro@unical.it; donatella.armentano@unical.it; emilio.pardo@uv.es

EXPERIMENTAL SECTION

All chemicals were of reagent grade quality. They were purchased from commercial sources and used as received.

Synthesis of UiO-66: UiO-66 was synthesized by solvothermal method with some modifications.1 First, $ZrCl_4$ (466 mg, 2 mmol) and H2BDC (332 mg, 2 mmol) were dissolved in DMF (50 ml, 646 mmol), and then the mixture solution was stirred for 30 min. Afterwards, the solution was transferred into a Teflon-lined stainless-steel autoclave and was placed in an oven and heated at 120℃ for 24 h. The batch-synthesized samples were collected by centrifugation (6000 rpm for 10 min), washed three times with fresh DMF (20 ml each time), and then with ethanol (20 ml each time). After ethanol washing, the samples were collected and oven-dried under vacuum at 100 ℃ for 16 h.

Synthesis of ZIF-8: To prepare ZIF-8, $Zn(NO_3)_2{\cdot}6H_2O$ (5950 mg, 20 mmol) and 2-methylimidazole (2-MeIm) (6157 mg, 75 mmol) were dissolved in methanol (150 mL, 3710 mmol) to obtain a uniform dispersion, respectively. Subsequently, the above solutions were mixed and stirred at room temperature for 24 h. The white precipitates were centrifuged, washed three times with fresh methanol (30 ml each time), and dried in vacuum oven at 60 ◦C for 24 h.

Preparation of UiO-66 and ZIF-8 based Photopolymer Resin Composite: To prepare the composite resin, MOF microcrystalline powder (4 g) was added to the commercial acrylate-based photopolymer resin (36 g) under continuous magnetic stirring at room temperature which corresponds to a filler loading of 10 wt.%. The selected 10 wt% MOF loading was established based on preliminary screening experiments evaluating formulations with 5 wt%, 10 wt%, and 25 wt% filler content. Our initial testing revealed that lower loadings (5 wt%) resulted in an insufficient density of active sites, yielding diminished dye adsorption capacities. Conversely, higher loadings (25 wt%) led to significant processing and structural drawbacks: the increased suspension viscosity and UV light scattering/attenuation during photopolymerization impaired layer-by-layer cure depth and interlayer cohesion. Furthermore, 25 wt% composites exhibited severe mechanical degradation (increased brittleness and micro-crack formation) due to poor interfacial adhesion and particle agglomeration during resin curing. Therefore, 10 wt% was identified as the optimal loading, offering the ideal compromise between preserving printability, structural integrity, and mechanical stability. The mixture was maintained under stirring in the dark for 48 h to ensure uniform dispersion and to prevent premature photopolymerization. The resulting filler loaded resin was directly used for SLA 3D printing without further modification.

Preparation of 3D Printed UiO-66 and ZIF-8 based Photopolymer Membranes: The neat resin (control sample) and filler loaded resin composites were fabricated into membrane structures using SLA printing technique with an Elegoo Mars P 3D printer (Elegoo Inc., Shenzhen, China). The membrane geometry was designed as a hexagonal array (dimensions: $50 \times 50 \times 1$ mm^3) characterized by a side length of 1.0 mm and separated by 0.4 mm-thick walls, using Chitubox slicing software. For MOF composites, layer-specific exposure times were applied to ensure optimal polymerization: for ZIF-8, 45 s per layer for layers 1–10 and 15 s for layers 11–20, while for UiO-66, 30 s per layer for layers 1–10 and 7 s for layers 11–20. After printing, the membranes were washed with isopropanol to remove uncured resin and post-cured under a 405 nm UV lamp (Unitak 3D Power UV lamp, model UniT083EU, 6 W) for 10 minutes to ensure complete polymerization and structural integrity.

Physical Techniques. Powder X-ray Diffraction (PXRD): XRD was used to determine the structural properties of the printed membranes using a Bruker D2 PHASER (second generation, Germany)

diffractometer equipped with Cu Kα radiation (λ = 1.54056 Å) over a 2θ range of 5–50°. Using a step size of 0.01° and a counting time of 1.0 seconds per step under ambient conditions. The X-ray tube operated at 40 kV and 40 mA.

Fourier Transform Infrared Spectroscopy (FTIR): For the analysis of chemical functional groups and confirmation of UiO-66 and ZIF-8 incorporation into the printed polymeric matrix, FTIR was conducted on an OMNIC Paradigm spectrometer. The samples were scanned in the wavenumber range of 4000–400 cm^{-1} with spectral resolution of 1 cm^{-1}.

Scanning Electron Microscopy (SEM): Samples were first mounted on aluminum stubs (25 mm) using conductive carbon tape and coated with a thin layer of conductive metal using a Quorum Q150T ES sputter coater (Quorum Technologies Ltd., UK). The coating process involved a (2M STRUMENTI) for QT pulse rod evaporation under vacuum to ensure uniform coverage. After coating, the samples were imaged using a Zeiss Gemini Crossbeam 350 SEM (Carl Zeiss AG, Germany) in secondary electron mode at an accelerating voltage of 15 kV. Images were collected under high vacuum conditions to observe the surface morphology.

Gas Sorption. N_2 adsorption isotherms at 77 K were conducted on the prepared crystalline MOF samples with a BELSORP MINI X instrument. MOFs were activated at 60 °C under reduced pressure (10–6 Torr) for 16 h prior performing the sorption measurements. The Brunauer-Emmett Teller (BET) surface areas were implemented from the N_2 adsorption isotherm data.

Energy Dispersive X-ray Spectroscopy (EDS): The membrane samples were mounted on aluminum stubs using conductive carbon cement and coated using a Quorum sputter coater. SEM and EDS analyses were performed at 15 kV using a ZEISS Gemini Crossbeam 350 SEM equipped with an EDS system at the Università della Calabria (Italy).

Confocal Laser Scanning Microscopy (CLSM): An optical microscope (Leica LAX, Leica Microsystems, Germany) was used to assess surface morphology and microstructure of printed membranes. In order to evaluate the quality of printing and the surface features, three-dimensional surface topography and roughness parameters were analyzed.

Mechanical characterization by nanoindentation and tensile testing: The synthesized membranes were tested using a constant strain rate protocol by undergoing dynamic nanoindentation in sinus mode under displacement control. Experiments were conducted on a nanoindentation platform (NHT, CSM Instruments, Switzerland) equipped with a spherical indenter tip of 20 µm radius and a maximum load capacity of 500 mN, at room temperature. The applied load ranged from 1 to 50 mN. In order to continuously measure the viscoelastic response of the materials, a sinusoidal perturbation was superimposed with an amplitude of 5 mN and a frequency of 5 Hz during loading. The strain rate was maintained at 0.05 s^{-1} to ensure uniform penetration. By using this setup, quasi-static mechanical parameters like hardness and reduced elastic modulus, as well as dynamic properties like storage modulus (E’) and loss modulus (E''), could be evaluated simultaneously. Uniaxial tensile tests were performed on dog-bone specimens fabricated by MSLA using the neat resin and the resins containing 10 wt% UiO-66 or 10 wt% ZIF-8. The tests were conducted at a constant crosshead speed of 1 mm/min using a universal testing machine (MTS, Criterion C42, USA). The gauge length of the specimens was 15 mm. The engineering stress was calculated from the applied load and the initial cross-sectional area measured for each specimen, whereas the axial strain was obtained by digital image correlation (DIC). Images were acquired at 15 frames/s using a Sony ICX625-based Prosilica GT 2450 camera with a

resolution of 2448 × 2050 pixels. The optical system consisted of a Linos Photonics objective and a Rodagon lens with a focal length of 80 mm. The tensile Young's modulus was determined from the slope of the initial linear portion of the engineering stress–strain curve. A reliable 0.2% offset yield strength could not be determined for the MOF-containing resins from the measured stress–strain curves. Therefore, the quantitative comparison of the tensile properties of the three formulations was restricted to Young's modulus. Three specimens were analyzed for the neat resin and the ZIF-8-containing resin, whereas two analyzable specimens were considered for the UiO-66-containing resin because some printed specimens exhibited visible manufacturing defects.

Dye Removal Experiment: The adsorption performance of the fabricated 3D printed membranes for the removal of organic dye was evaluated at room temperature in a series of experiments. The adsorption experiments were deliberately conducted at ambient pH ~5.0, (unbuffered aqueous solution), which corresponds to the typical pH of natural surface and deionized water, contaminated by traces of dye (5 ppm), avoiding the need for artificial buffer additives or chemical pH adjustments during practical remediation. In batch capture experiments, dye solutions with known concentrations were used to evaluate adsorption performance. UV–vis spectroscopy (Varian Cary 50 spectrophotometer) at wavelength ranges of 300-800 nm was used to establish calibration curves for dyes. Evaluating BG capture at 5 ppm and ambient pH ~5.0 served as a functional proof-of-concept to confirm that the embedded MOF fillers remain active and accessible within the photopolymerized matrix. BG was used as a model dye to assess the potential 3D-printed resin membranes' adsorption capabilities. Each experiment involved immersing a membrane sample of 200 mg (cut into squares) in a 50 ml of an aqueous solution of BG dye (5 ppm) with continuous shaking. We monitored dye removal after 72 h of contact. An aliquot of the sample was monitored at selected time intervals (0, 5, 10, 20, 30, 60, 120, 180, 240, 300, 360, 480, and 1080) and was examined using spectrophotometer. The maximum absorbance wavelength was at 625 nm. The dye removal efficiency (R) was also calculated using the equation below to assess adsorption extent:

$$R (\%) = ((C_o-C_t))/C_o \times 100$$

We conducted these experiments in triplicates to ensure reproducibility, and no filtration was required since the solid membranes were easily retrieved after each test with tweezers.

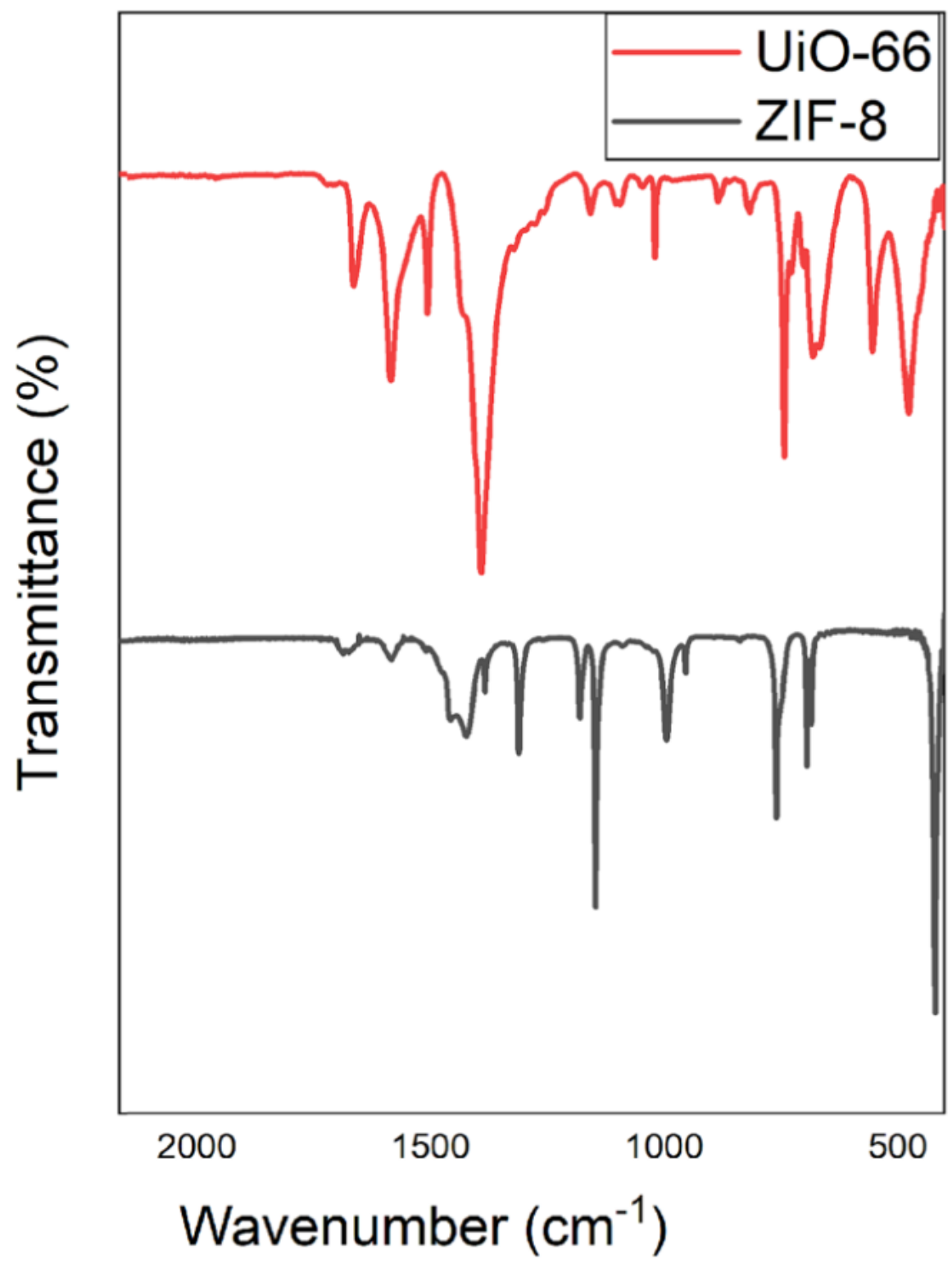

Fig. S1. FTIR spectra of ZIF-8 and UiO-66 fillers. (To maintain complete clarity and scientific rigor, we have retained the full wavenumber range in Fig. 3 of the main manuscript to provide the overall spectral profile of the samples. However, to facilitate a clearer, more detailed interpretation of the key vibrational bands, we have added here zoomed-in spectra focusing on the region up to 2000 cm-1).

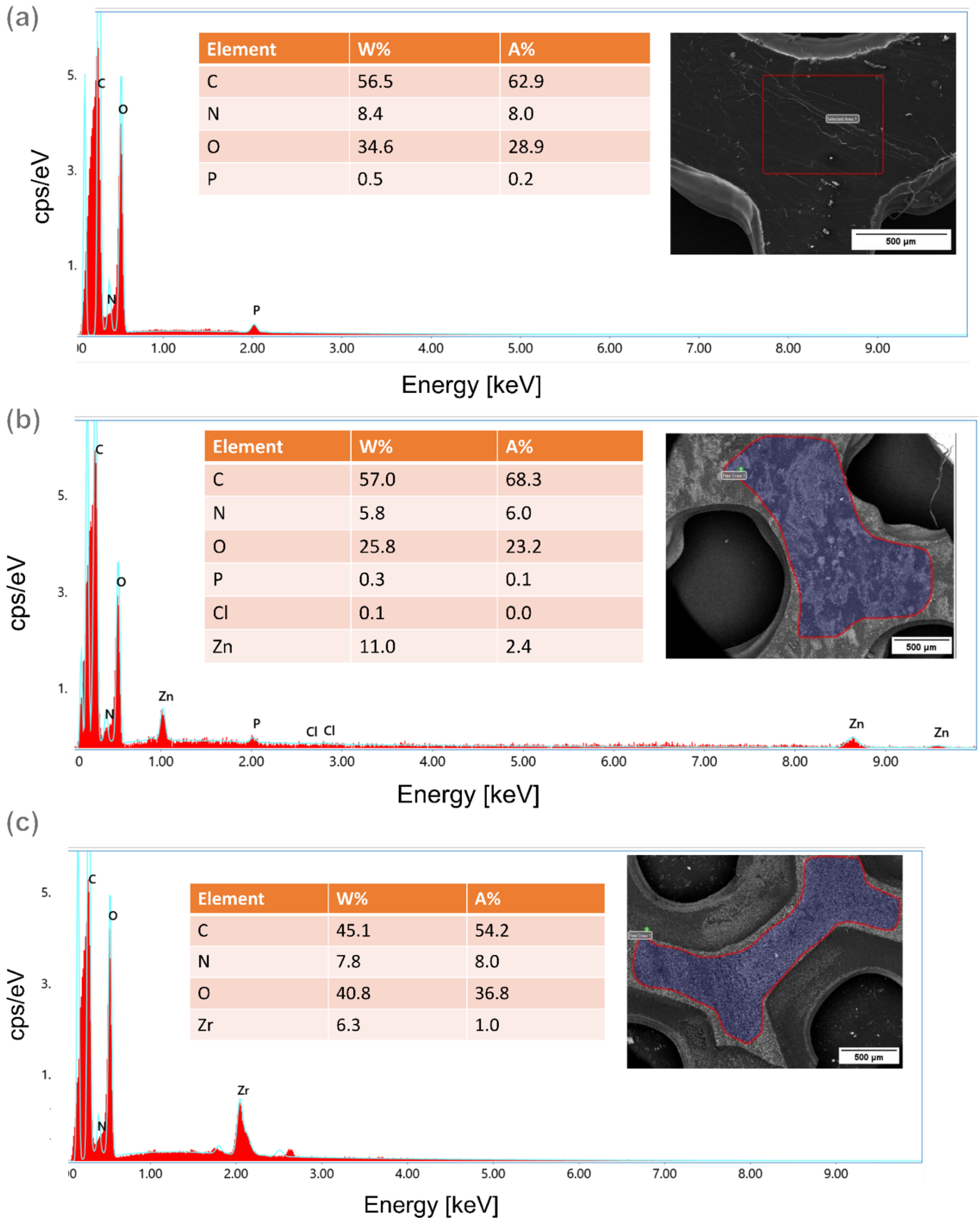

(a)

Element	W%	A%
C	56.5	62.9
N	8.4	8.0
O	34.6	28.9
P	0.5	0.2

(b)

Element	W%	A%
C	57.0	68.3
N	5.8	6.0
O	25.8	23.2
P	0.3	0.1
Cl	0.1	0.0
Zn	11.0	2.4

(c)

Element	W%	A%
C	45.1	54.2
N	7.8	8.0
O	40.8	36.8
Zr	6.3	1.0

Fig. S2. The EDS spectra of (a) pristine resin membrane, (b) ZIF-8-Resin, and (c) UiO-66-Resin.

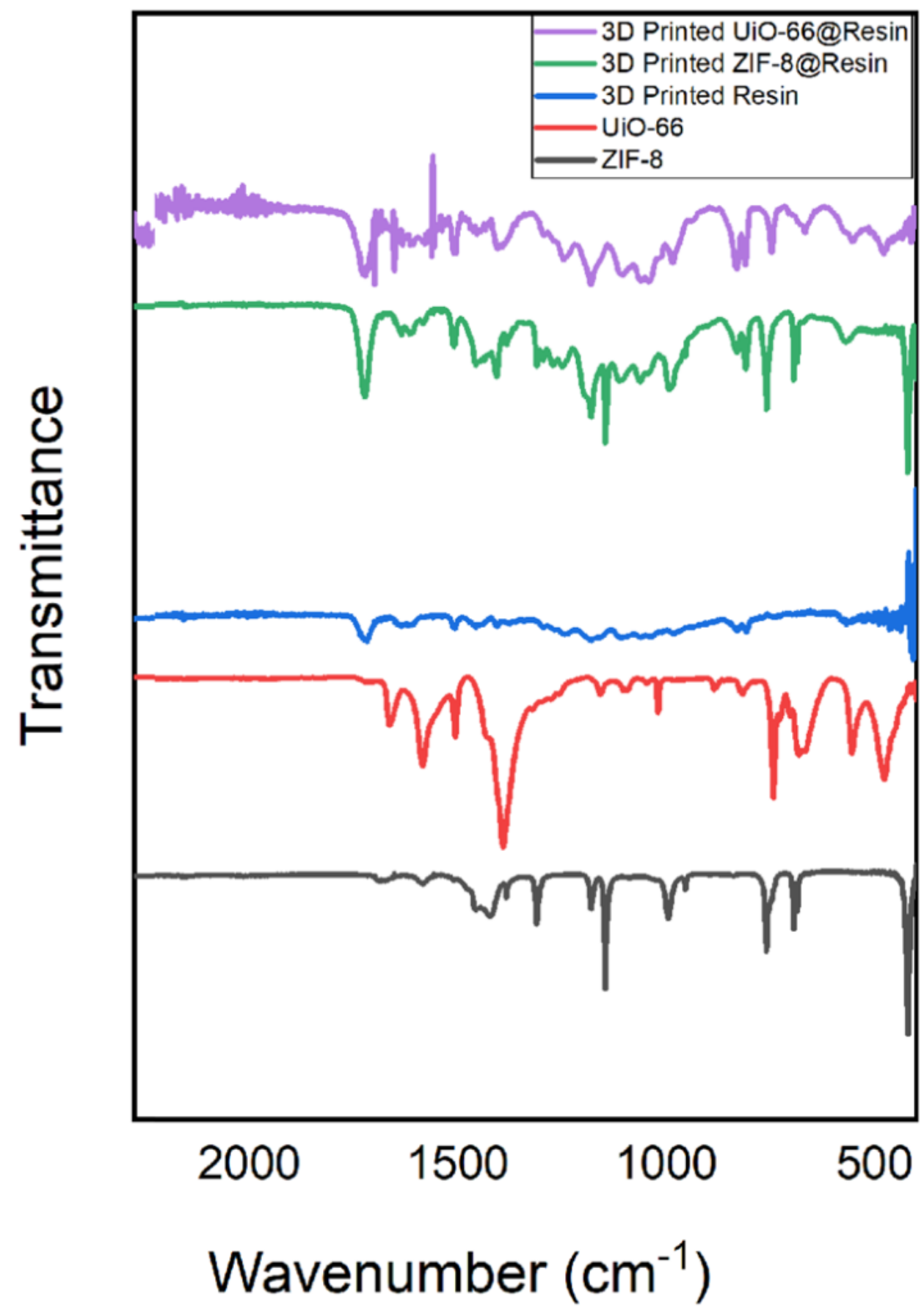

Fig. S3. FTIR spectra of MOF powders and 3D printed membranes. Patterns include: ZIF-8 powder (Black), UiO-66 (Red), 3D printed resin (Blue), 3D printed ZIF-8-Resin (Green), and 3D printed UiO-66-Resin (Purple). (To maintain complete clarity and scientific rigor, we have retained the full wavenumber range in Fig. 8 of the main manuscript to provide the overall spectral profile of the samples. However, to facilitate a clearer, more detailed interpretation of the key vibrational bands, we have added here zoomed-in spectra focusing on the region up to 2000 cm-1).

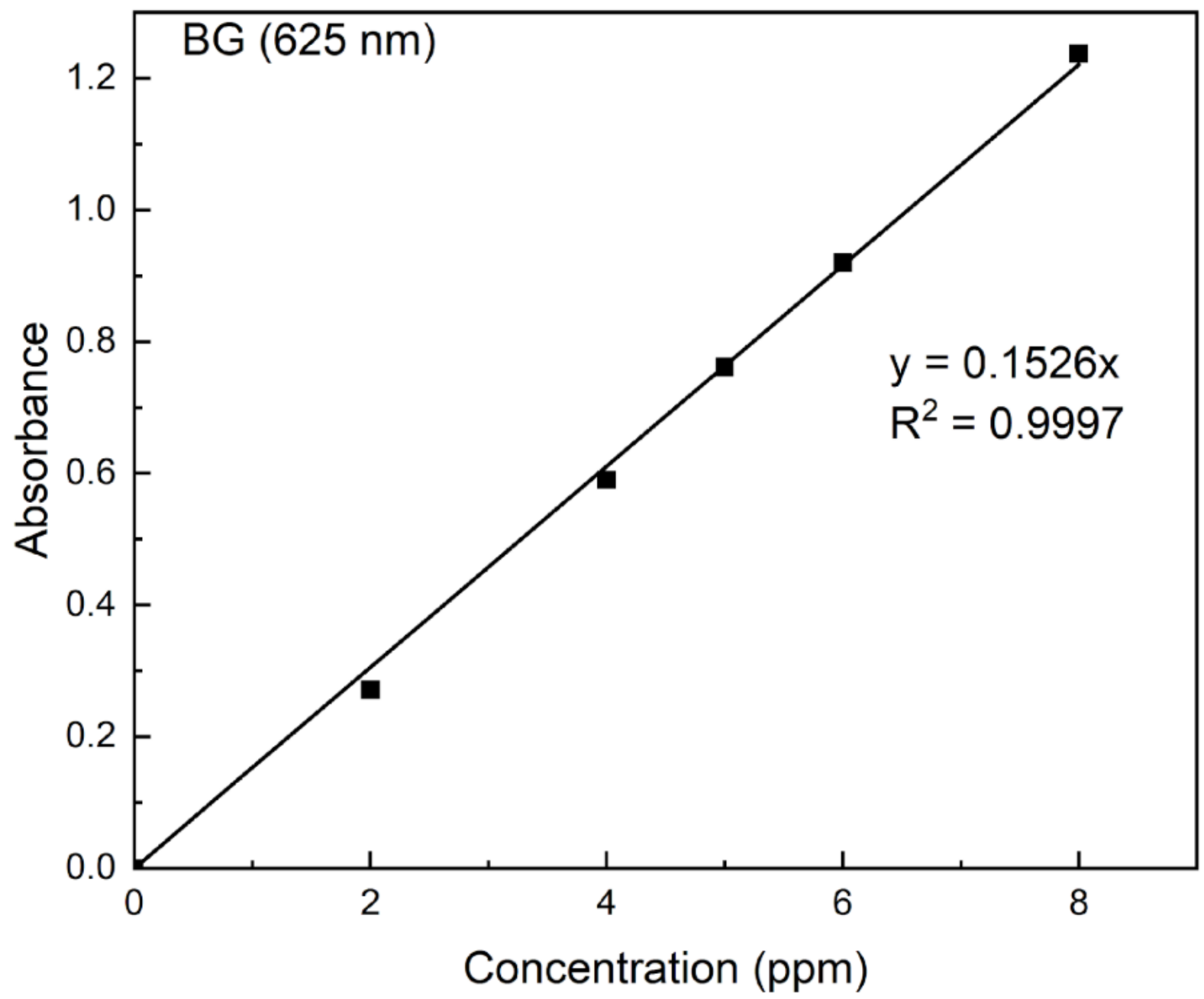

Fig. S4. Calibration curve of BG dye based on UV–vis absorbance measurements at 300-800 nm.

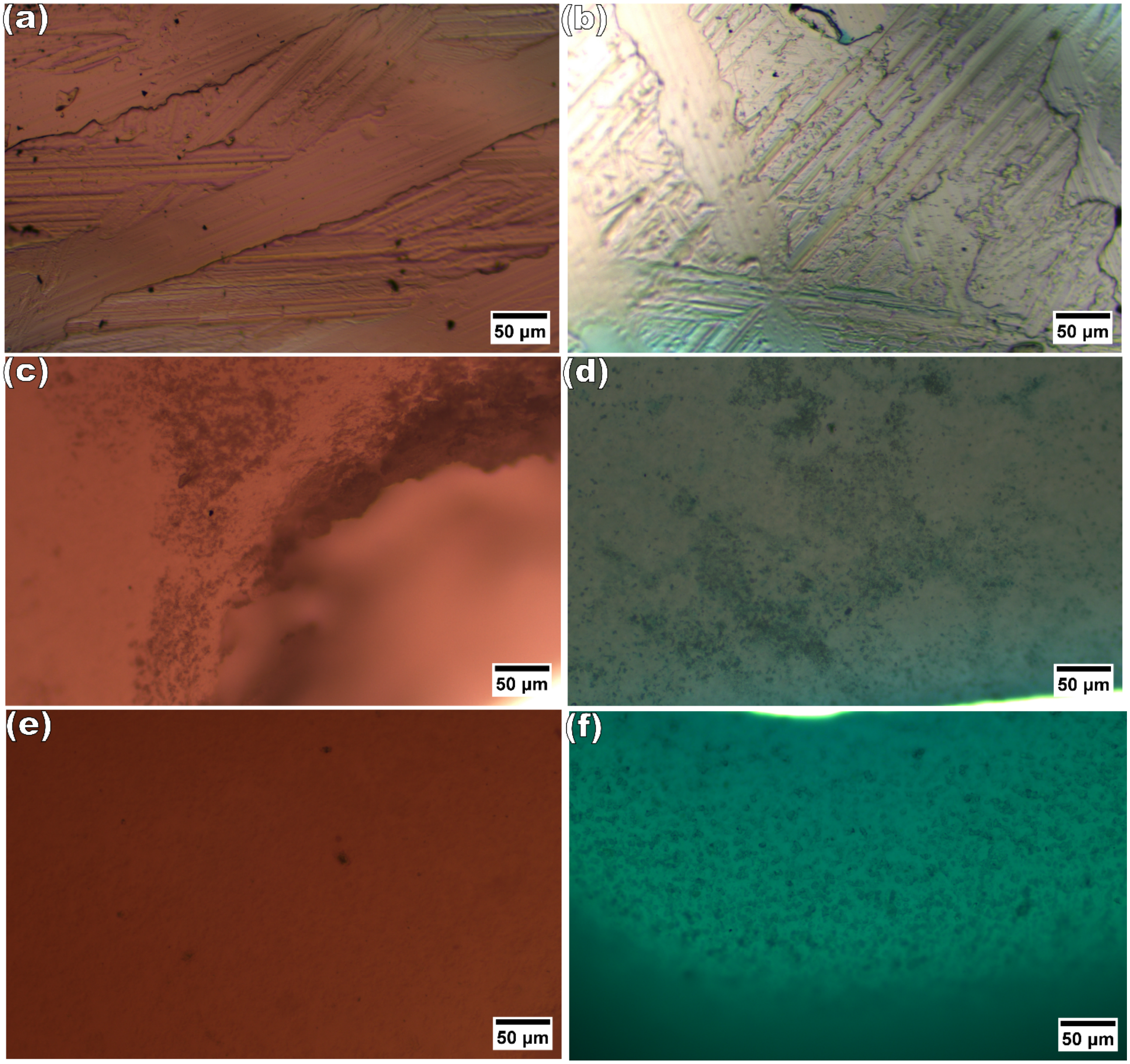

Fig. S5. Digital images of 3D printed membranes before and after 24 h of BG dye. (a, b) 3D printed resin membrane, (c, d) 3D printed ZIF-8-resin membrane, and (e, f) 3D printed UiO-66-resin membrane.

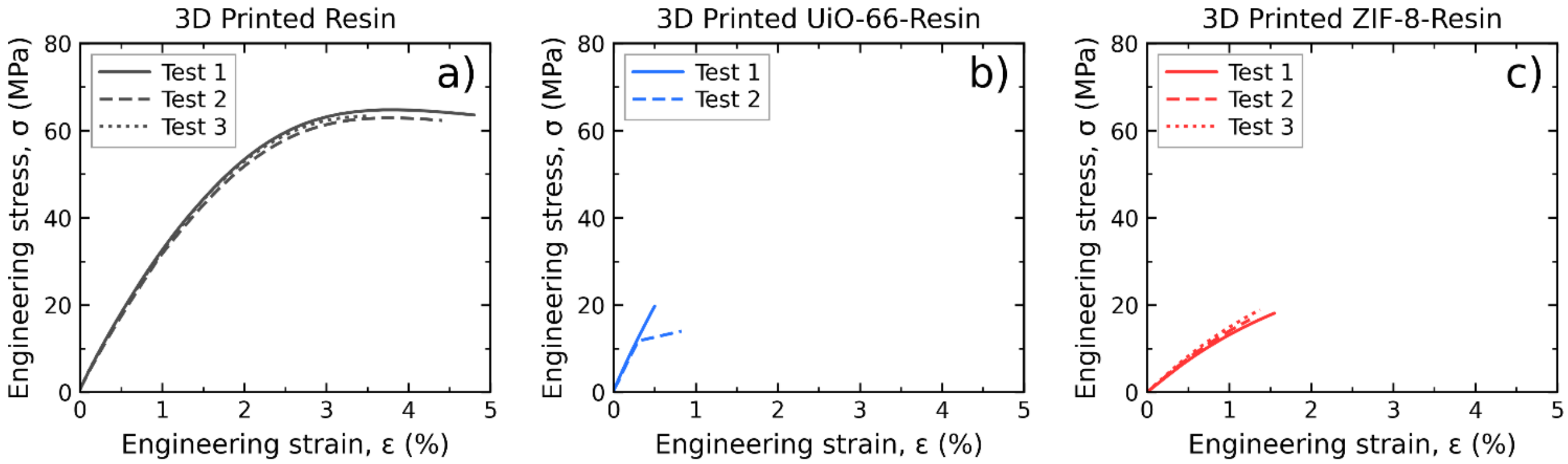

Fig. S6. Engineering stress–strain curves obtained from uniaxial tensile tests on MSLA-printed dog-bone specimens of (a) neat resin, (b) resin containing 10 wt% UiO-66, and (c) resin containing 10 wt% ZIF-8.

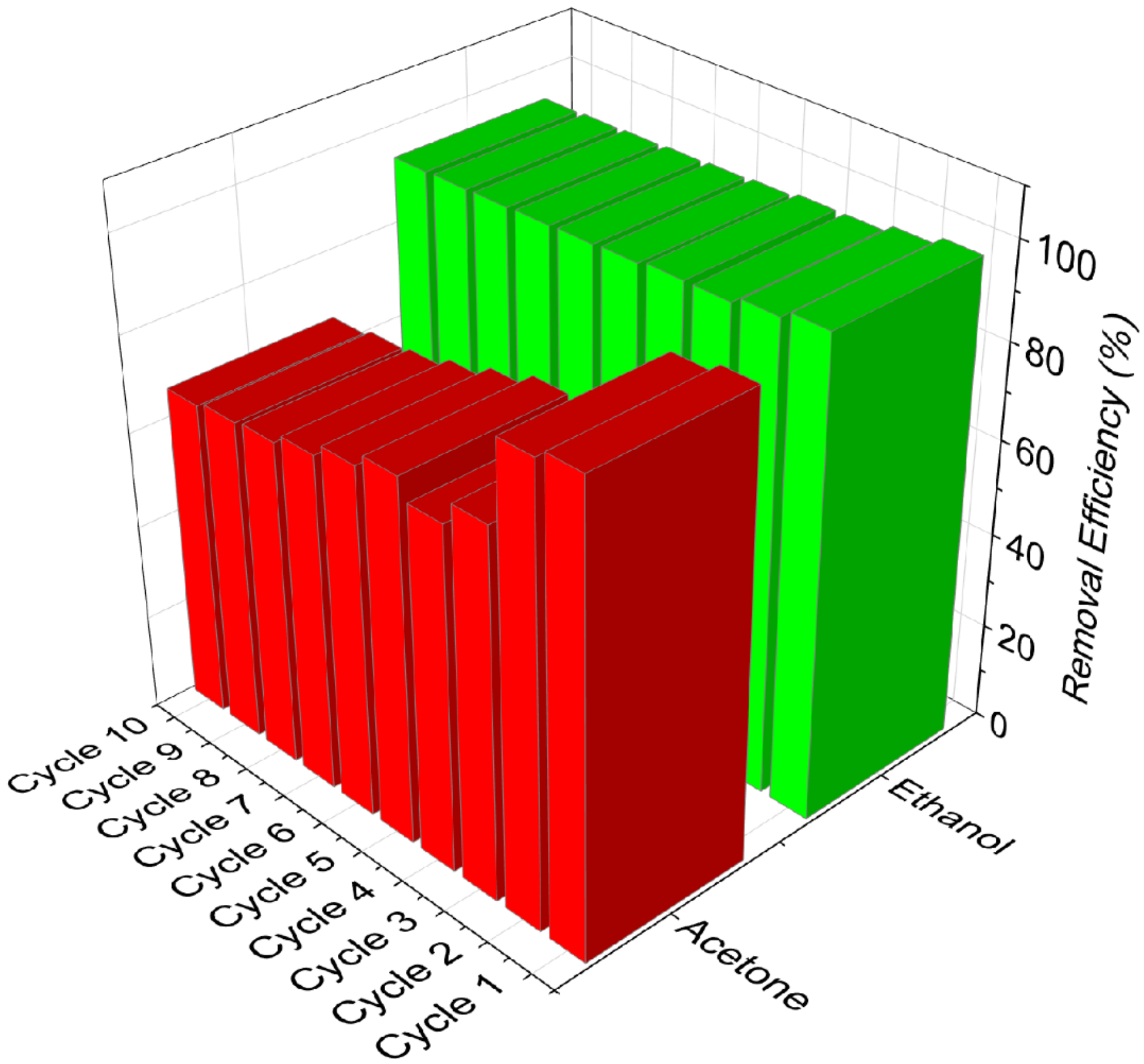

Fig. S7. Regeneration performance of 3D printed ZIF-8-resin membrane for BG removal (5 ppm, 50 mL) over ten multiple cycles. Comparison between washing with acetone and ethanol as regenerants.

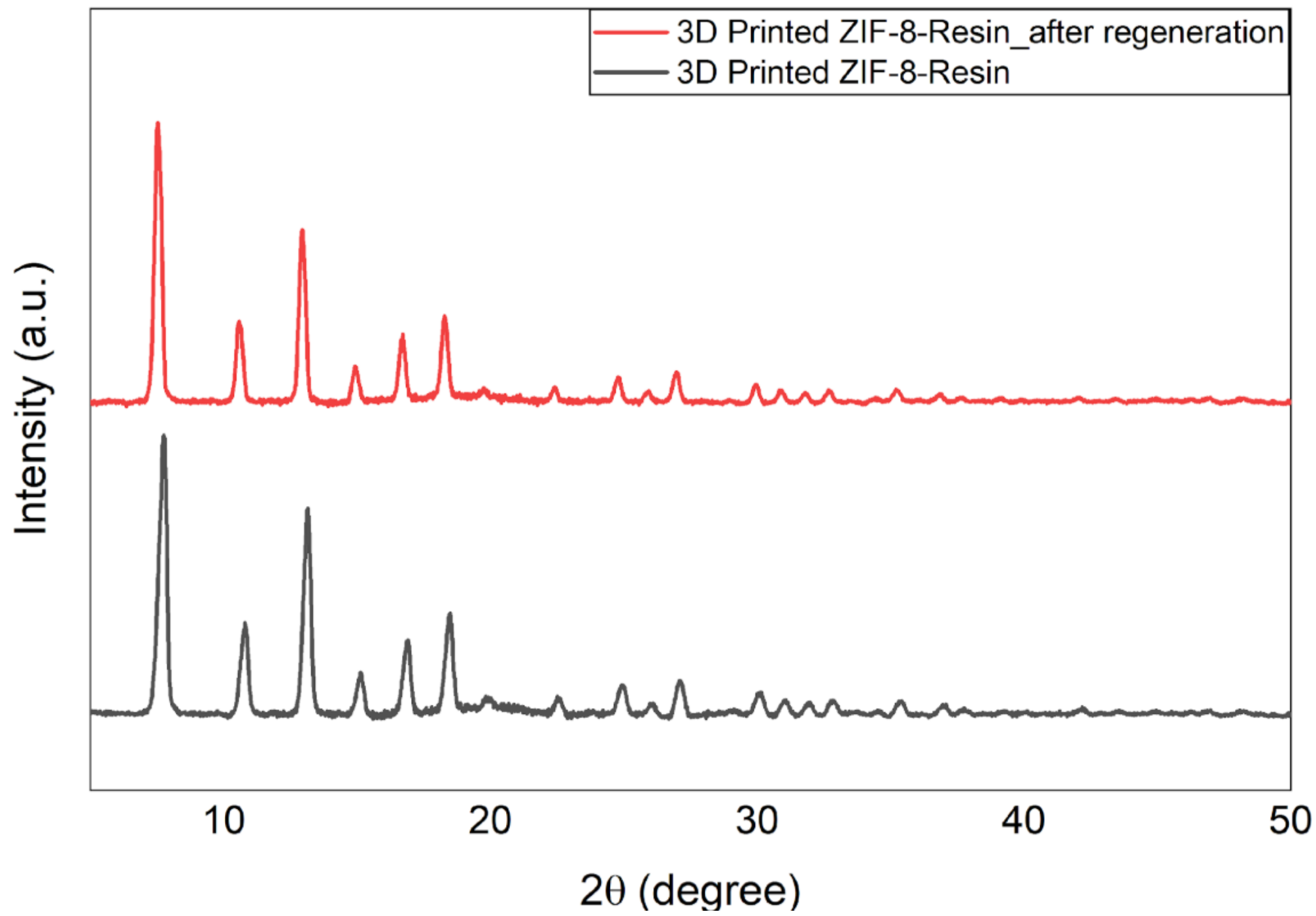

Fig. S8. PXRD patterns of the 3D printed ZIF-8-resin after five adsorption–desorption regeneration cycles (Red) in comparison with the synthesized 3D printed ZIF-8-resin before use.

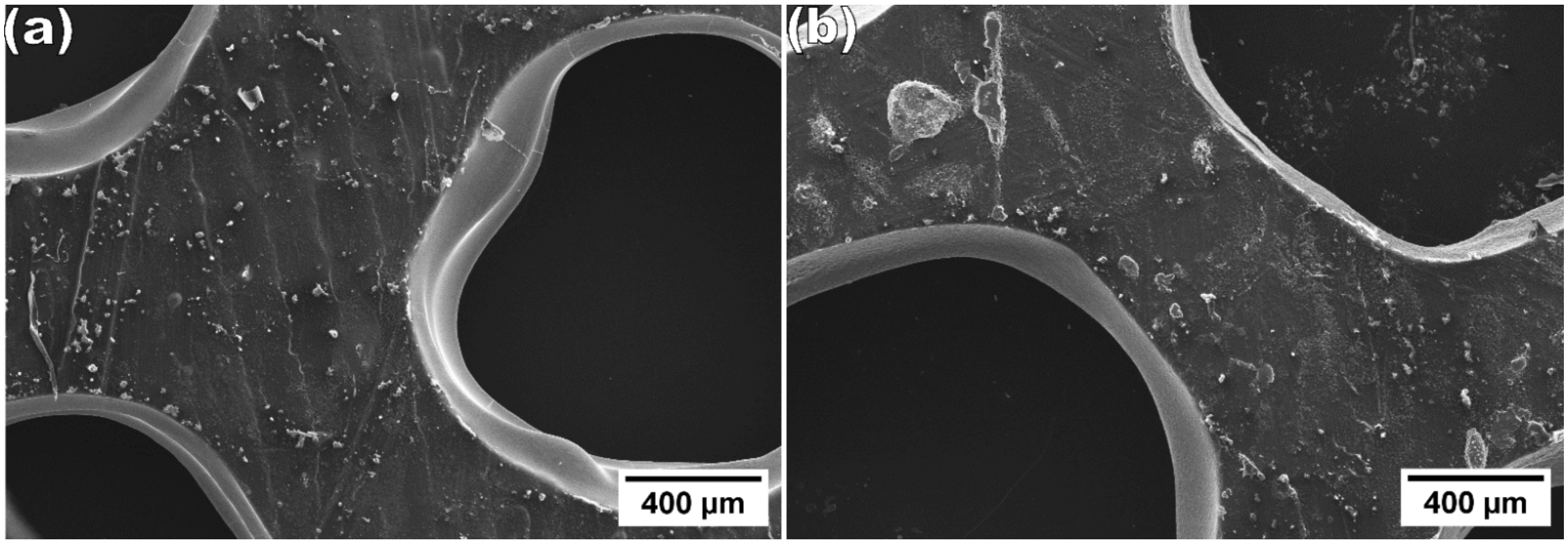

Fig. S9. SEM images for 3D-printed ZIF-8-resin membrane top surface (a, b) before and after regeneration tests, respectively.

Table S1. BET surface area and Pore Volume for the activated MOFs.

MOFs	BET surface area (m^2g^{-1})	Average pore width (nm)	Total Pore volume (cm^3g^{-1})
ZIF-8	1463.10	0.77	0.6389
UiO-66	863.96	0.91	0.3579

Table S2. Time-dependent removal efficiency (R%) of 3D-printed membranes based on pristine resin, ZIF-8–resin, and UiO-66–resin towards BG dye at an initial dye concentration of 5 ppm. Capture experiments were conducted in triplicate, and results are reported as average values ± 3 SD.

Time (min)	**3D Printed Resin (R%)**	**3D Printed ZIF-8-Resin (R%)**	**3D Printed UiO-66-Resin (R%)**
0	0.71	9.54	2.28
5	1.27	15.11	7.28
10	2.41	21.58	7.39
20	2.81	31.69	15.58
30	3.18	66.27	19.80
60	3.95	70.47	24.68
120	4.80	73.82	25.42
180	5.60	79.95	27.45
240	6.39	81.20	27.13
300	7.15	82.748	32.47
360	8.49	89.93	36.55
480	8.66	96.91	36.69
1080	8.77	97.96	38.94

Table S3. Kinetics models of the adsorption process.*

Membrane	Kinetic Model	R^2	Rate Constant (k)	Calculated Qe (mg/g)
**Pristine Resin**	PFO	0.779	$k_1 = 0.00397\ min^{-1}$	0.38
	PSO	0.940	$k_2 = 4.6937\ g{\cdot}mg^{-1}{\cdot}min^{-1}$	0.0175
	ID	0.857	$k_{id} = 0.01633$	C = 0.03488
**ZIF-8-Resin**	PFO	0.951	$k_1 = 0.00925\ min^{-1}$	1.10
	PSO	0.749	$k_2 = 0.81012\ g{\cdot}mg^{-1}{\cdot}min^{-1}$	0.0257
	ID	0.977	$k_{id} = 0.0908$	C = –0.07733
**UiO-66-Resin**	PFO	0.903	$k_1 = 0.00371\ min^{-1}$	0.596
	PSO	0.865	$k_2 = 3.50707\ g{\cdot}mg^{-1}{\cdot}min^{-1}$	0.00946
	ID	0.966	$k_{id} = 0.02273$	C = –0.00268

*The adsorption process has been modelled by using different adsorption kinetics models: the linearized forms of the Lagergren's pseudo-first order rate expression (PFO) and of the pseudo-second order rate expression (PSO),[3,4] and the intraparticle diffusion adsorption kinetics model (ID).[5] OriginPro2024 software was used to fit experimental adsorption data as a function of time.[6,7] K = calculated adsorption rate constants; Qe = equilibrium loading; R^2 = coefficient of determination at initial BG concentrations of 5 ppm.

Table S4. Regeneration of 3D printed ZIF-8-Resin membrane up to five an then ten adsorption–desorption cycles in term of Removal Efficiency (%).

3D Printed Membranes	R% I Cycle		R% II Cycle		R% III Cycle		R% IV Cycle		R% V Cycle	
	Acetone	Ethanol	Acetone	Ethanol	Acetone	Ethanol	Acetone	Ethanol	Acetone	Ethanol
**3D printed ZIF-8-Resin**	96.2	99.3	94.2	97.7	77	96.2	72.1	96.2	76.3	95.4

3D Printed Membranes	R% VI Cycle		R% VII Cycle		R% VIII Cycle		R% IX Cycle		R% X Cycle	
	Acetone	Ethanol	Acetone	Ethanol	Acetone	Ethanol	Acetone	Ethanol	Acetone	Ethanol
**3D printed ZIF-8-Resin**	73.4	95	70.6	94.7	68.4	94.4	67.7	94.2	66.5	94

Table S5. Tensile Young's modulus of MSLA-printed neat resin and composites containing 10 wt% UiO-66 or ZIF-8. Values are reported as mean ± standard deviation.

Formulation	Number of specimens, n	Young's modulus (GPa)
**Neat resin**	3	3.11 ± 0.06
**Resin/UiO-66**	2	3.83 ± 0.01
**Resin/ZIF-8**	3	1.54 ± 0.10

References

1- Zhao, J.; Xie, H.; & Meng, X. Tailoring Defect Density via Ultraviolet Illumination in UiO-66 for Efficient Photocatalytic Nitrogen Fixation. *Langmuir* **2025**, *41*, 31559-31568.

2- Liu, J.; Zhong, X.; Gong, X.; Deng, L.; Tan, G.; Zhang, Q.; Xiao, Z.; Yao, Q.; Liu, S.; Gao, Y.; Wang, L.; Lu, L. Highly Sensitive Turn-On Electrochemical Sensing of Organophosphorus Pesticides by Integration of Homogeneous Reaction and Heterogeneous Catalytic Signal Amplification. *Food Chem.* **2024**, *458*, 140275.

3- Ho, Y.S; McKay, G. Pseudo-second order model for sorption processes, *Process Biochemistry*, **1999**, 34, 5, 451-465.

4- Revellame, E. D.; Fortela, D. L.; Sharp, W.; Hernandez, R.; Zappi, M. E. Adsorption kinetic modeling using pseudo-first order and pseudo-second order rate laws: A review, *Cleaner Engineering and Technology* **2020**, *1*, 100032.

5- Wang, J.; Guo, X. Rethinking of the intraparticle diffusion adsorption kinetics model: Interpretation, solving methods and applications, *Chemosphere*, **2022**, *309*, 136732.

6- OriginPro 2024, Version 10.1; OriginLab Corporation: Northampton, MA, USA.

7- Belhachemi, M.; Addoun, F. Comparative Adsorption Isotherms and Modelling of Methylene Blue onto Activated Carbons. *Appl. Water Sci*. **2011**, *1* (3−4), 111−117.
